# Long‐Term Effects of Orlistat on Lipid Metabolism and Anthropometric Indices: A Meta‐Analysis of Clinical Trials

**DOI:** 10.1155/jobe/9068305

**Published:** 2026-02-23

**Authors:** Alireza Khodadadiyan, Yalda Khazraei, Maliheh Kamali, Kimiya Kolaei, Parmida Aminzadeh, Golnaz Yazdanpanah, Ali Shams, Maryam Feili, Melika Ghaffari, Mehrasa Hosseini, Mehdi Bazrafshan, Hamed Bazrafshan drissi, Alireza Arzhangzadeh

**Affiliations:** ^1^ Cardiovascular Research Center, Shiraz University of Medical Sciences, Shiraz, Fars, Iran, sums.ac.ir; ^2^ School of Medicine, Shiraz University of Medical Sciences, Shiraz, Fars, Iran, sums.ac.ir; ^3^ Cardiovascular Research Center, Shiraz University of Medical Sciences, Shiraz, Fars, Iran, sums.ac.ir

**Keywords:** anthropometric indices, lipid profile, lipid-lowering drugs, nutrition, obesity, orlistat

## Abstract

**Background:**

Orlistat is a potent lipase inhibitor utilized as a preventive agent for obesity and fat absorption control. Existing literature presents conflicting findings regarding its impact on lipid parameters.

**Methods:**

This systematic review followed the PRISMA guidelines and was registered in PROSPERO (ID: CRD42024550889). A comprehensive search of PubMed, Scopus, Web of Science, and Cochrane Register of Controlled Trials was conducted for studies published before January 19, 2025. Eligible studies included randomized controlled trials (RCTs) evaluating orlistat in adults (≥ 18 years) with dyslipidemia. Furthermore, the Grading of Recommendations, Assessment, Development, and Evaluations assessment tool was employed to analyze the certainty of evidence or each outcome.

**Results:**

A total number of 1369 participants, with 682 in treatment and 687 in control categories, were included in our study. Orlistat reduced body mass index (BMI) (SMD [95% CI]: −0.30 [‐0.58, −0.03], *p* value (heterogeneity) = 0.026), and also it was associated with a decrease in high‐density lipoprotein cholesterol (SMD (95% CI): −0.31 [‐0.48, −0.13], *p* value (heterogeneity) = 0.436). Changes in waist circumference (WC) and triglycerides (TGs) did not reach statistical significance in the primary analysis (WC: SMD [95% CI] −0.1562 [‐0.3138; 0.0015], *I* ^2^ = 0.0%, *p*‐value (heterogeneity) = 0.7572; TG: SMD [95% CI] −0.1668 [‐0.7979; 0.4642], *I* ^2^ = 97.7% *p* value (heterogeneity) < 0.0001); however, after publication‐bias adjustment using the trim‐and‐fill sensitivity analysis, meaningful reductions were discovered for WC (SMD (%95CI): −0.1712 [‐0.3248; −0.0176], *I* ^2^ = 0.0%, *p* value (heterogeneity) = 0.7696) and TG (SMD (%95CI): −0.8900 [‐1.6619; −0.1181], *I* ^2^ = 97.9%, *p* value (heterogeneity) < 0.0001). The secondary analysis demonstrated that follow‐up duration accounted for 30% of TG heterogeneity, suggesting a small but significant decline in orlistat’s TG‐lowering effect over time (slope: −0.1239; 95% CI: −0.2355, −0.0123; *p* value = 0.0295). No significant changes were observed in other parameters of the study. Besides, gastrointestinal issues were the most frequently reported adverse events among the studies.

**Conclusion:**

Our findings suggest that orlistat meaningfully reduces BMI but is associated with decreased HDL‐C, which may be undesirable given HDL‐C’s protective role in cardiovascular health. Evidence for reductions in TG and WC is uncertain: the primary meta‐analysis showed no statistically significant effects, whereas trim‐and‐fill sensitivity analysis suggested potential reductions. No significant short‐term impact on TG was observed, though a modest reduction may emerge with prolonged use.

## 1. Introduction

Cardiovascular disease (CVD) remains one of the leading causes of morbidity and mortality worldwide, with dyslipidemia being a major modifiable risk factor [1, 2]. In 2021, the World Heart Federation found that CVDs caused around 20 million deaths, a significant increase from 12.1 million in 1990 [3]. Also, it has been demonstrated that ischemic heart disease is the leading cause of premature death in 146 countries for men and 98 countries for women [3]. Considering the latest advancements in preventive and lipid‐lowering therapies, such as the widespread use of statins, a substantial residual risk of dyslipidemia as a major CVD risk factor persists. Dyslipidemia is a medical term that refers to an imbalance of lipids within the bloodstream [1, 4]. It can be primary, caused by genetic mutations, or secondary, resulting from lifestyle factors and other medical conditions, like obesity, diabetes, hypothyroidism, and family history [5, 6]. Obesity, known for its adverse lipid profiles, often compounds with an elevated level of triglyceride (TG) and low‐density lipoprotein (LDL) and a decreased amount of high‐density lipoprotein (HDL) [7].

Orlistat is a potent gastrointestinal lipase inhibitor used as a preventive agent for obesity and fat absorption control. Orlistat functions by forming a covalent bond with the active serine residue in gastric and pancreatic lipases, the enzymes that are crucial for breaking down TGs within the digestive system. Clinical trials indicate that orlistat, combined with diet and exercise, results in 2–3 kg (4–7 lb) more weight loss over 1 year compared to lifestyle changes alone [8, 9]. Likewise, some meta‐analyses discovered that long‐term use may reduce systolic and diastolic blood pressures by 2.5 mmHg and 1.9 mmHg, respectively [10, 11].

Moreover, several studies show that combining orlistat with lipid‐lowering drugs like ezetimibe, fenofibrate, and statins has notably interesting synergistic effects, leading to greater reductions in LDL‐C and small dense LDL‐C levels in overweight and obese hypercholesterolemic patients compared to using each medication alone [12–14]. The combination also led to significant reductions in body mass index (BMI), insulin resistance, serum uric acid, and plasma lipoprotein‐associated phospholipase A2 activity [13]. Also, it has been shown that in a case of combined hyperlipidemia with predominant hypertriglyceridemia, a combination of gemfibrozil and orlistat was effective in reducing serum TG levels where each drug alone was not [13]. In turn, lipid profile optimizations can lead to notable changes in anthropometric indices, including waist circumference (WC) in patients.

Our literature presents inconsistent findings regarding the impact of orlistat on various lipid profile parameters. Findings from a meta‐analysis in 2017 indicated that orlistat administration has a substantial positive impact on high‐density lipoprotein cholesterol (HDL‐C), LDL‐C, total cholesterol (TC), and TG [15]. Furthermore, while several reports indicated its significant impact on TG, LDL, HDL, TC, and BMI, others indicated only modest or nonsignificant changes in these variables [12, 13, 15, 16]. Furthermore, our study is the first analysis investigating apolipoprotein B, and waist‐to‐hip ratio (WHR), as well as the aforementioned lipid parameters. Apolipoprotein B (ApoB) provides a more precise indication of the number of atherogenic particles, particularly small dense LDL, therefore offering a stronger prediction of cardiovascular risk than LDL‐C alone. Similarly, while BMI is a general index of body mass (which does not differentiate lean muscle mass from adipose tissue), the WHR serves as a superior indicator of visceral fat accumulation and central obesity, which are more specific indicators of cardiovascular risk. Not only that, but by critically appraising the available data, this study seeks to provide insights that could inform future clinical practice and research.

## 2. Methods

This systematic review was conducted following the Preferred Reporting Items for Systematic Review and Meta‐analysis (PRISMA) statement and was registered at PROSPERO (International prospective register of Systematic Reviews, ID= CRD42024550889) [17].

### 2.1. Databases and Search Strategy

The computerized PubMed, Scopus, Web of Science, and Cochrane Register of Controlled Trials databases were systematically searched for relevant studies. Our search encompassed the studies published before the January 19, 2025, using the strategy provided in the Supporting File. The search strategy was modified appropriately for each database.

### 2.2. Population

The target population consisted of adults (age ≥ 18 years) with a BMI of ≥ 27 kg/m^2^ and confirmed dyslipidemia, defined as fasting TGs > 150 mg/dL or LDL > 150 mg/dL or TC > 200 mg/dL or HDL < 40 mg/dL for men or < 50 mg/dL for women. No limitations were considered regarding sex, nationality, type of CVD, time from onset, or medication in use.

### 2.3. Intervention

Our concentration was on the studies investigating on the effect of orlistat with or without lipid‐lowering drugs.

### 2.4. Comparator

The studies using placebo or continuing the standard treatment as a comparison group were considered. The presence of the control group was not limited.

### 2.5. Outcome

As the primary outcome, we include those studies discussing blood parameters, including LDL‐C, HDL‐C, TC, TGs, apolipoprotein B, and anthropometric indices like BMI, WC, and WHR. Additionally, the secondary prevention of interest was the occurrence of adverse events. The inclusion and exclusion criteria are listed as follows:

### 2.6. Inclusion Criteria

1. Randomized controlled trial (RCTs) studies
2. All studies evaluated orlistat as a standalone treatment or in combination with other pharmacotherapies.
3. Adults with established cardiovascular risk factors and a BMI ≥ 27 kg/m^2^
4. Patients exhibited at least one of the following lipid profile abnormalities: TGs > 150 mg/dL or LDL > 150 mg/dL or TC > 200 mg/dL or HDL < 40 mg/dL for men or < 50 mg/dL for women.

### 2.7. Exclusion Criteria

1. Studies without the assessment of orlistat‐related alterations in lipid parameters.
2. Studies that were published in languages other than English.
3. Population was not human.
4. Review studies, book chapters, letters to the editor, conference papers, observational cohort studies, cross‐sectional studies, and case series/reports.

### 2.8. Study Selection

After the automatic removal of the duplicate studies using the tools provided by the 20^th^ version of Endnote, reviews, editorials, letters to the editor, theses, abstracts, case series, and case reports were excluded; peer‐reviewed studies with interventional design, including RCTs and quasi‐experimental studies evaluating the effect of adding orlistat to lipid‐lowering drugs, including statins, were included. Two reviewers (AKh and MF) independently screened the title and abstract for relevant studies, and then another reviewer (YK) screened the full text of the studies, considering the eligibility criteria. Both screening rounds were conducted under the fourth reviewer’s (KK) supervision.

### 2.9. Risk of Bias Assessment

Two reviewers separately evaluated the risk of bias in the studies (AKh and PA). To assess the selection bias, performance bias, detection bias, attrition bias, reporting bias, and other possible biases, the Cochrane Collaboration’s assessment tool was employed [18]. The third reviewer (MB) performed as a supervisor if a consensus could not be reached.

### 2.10. Data Extraction

The data extraction form included the study’s first author, publish year, study design, the total number of participants and divided numbers as intervention and control arms, the total and divided age of intervention and control group (mean ± standard deviation), the sex of the participants, their history of CVD and medication, patients weight before and after the intervention, BMI, WC, WHR, the type of lipid‐lowering drugs and the dosage used, the therapy duration and follow‐up, probable second intervention, side effects, the primary outcome and measure, and ultimately, the mean and standard deviation of LDL‐C, HDL‐C, TC, TGs, as lipid profile measures, and the mean and standard deviation of apolipoprotein B before and after the intervention. Furthermore, two researchers (MK, KK) were assigned to extract the required data blindly, and any inconsistency was addressed through discussion with a third researcher (GY). Besides, inter‐reviewer agreement for categorical extraction items was assessed using Cohen’s kappa (*κ*). To quantify inter‐reviewer reliability for continuous data extraction, we calculated the intraclass correlation coefficient (ICC) using a two‐way random‐effects model with absolute agreement (ICC(2,1)) on paired extracted cells. ICCs are reported with 95% confidence intervals estimated by bootstrap resampling.

### 2.11. Data Analysis

Review Manager (RevMan) version 5.3 (Nordic Cochrane Centre, The Cochrane Collaboration, Copenhagen, Denmark) was utilized to analyze the data. Meta‐analysis was run via R programming version 4.4.2. Standardized mean difference (SMD) was considered the effect size for comparing the mean change of variables between the placebo and intervention groups. The I‐squared statistic (*I* ^2^), Tau‐squared (*τ* ^2^) statistics, and Cochran’s *Q* test indicated the amount of heterogeneity in the studies. The random effect model was used. Also, the Forest and Funnel plots were provided for each outcome, and Begg’s and Egger’s tests, and trim‐and‐fill method (Duval and Tweedie’s method) were applied to analyze the publication bias [19]. The *p*‐value was compared with a 0.05 significance level. Also, the 95% confidence interval was provided for the bias value.

Likewise, a meta‐regression was performed as secondary analysis following subgroup analysis if heterogeneity existed for outcomes. Follow‐up duration was included as a continuous moderator to assess its influence on treatment effects. The restricted maximum likelihood (REML) method was used to estimate between‐study variance (*τ* ^2^). The proportion of heterogeneity explained by the moderator was quantified using *R* ^2^, and statistical significance was determined using a *Z*‐test.

We applied the Grading of Recommendations, Assessment, Development, and Evaluations (GRADE) approach to evaluate the certainty of the evidence [20, 21]. Five domains were considered for downgrading or upgrading the evidence: risk of bias, inconsistency, indirectness, imprecision, and publication bias. The overall certainty of evidence was categorized into four levels: high, moderate, low, or very low. Two authors (HB, AA) performed the evaluations blindly, which was then followed by discussions with a third author (MH), if any inconsistency appeared.

## 3. Results

**FIGURE 1:**
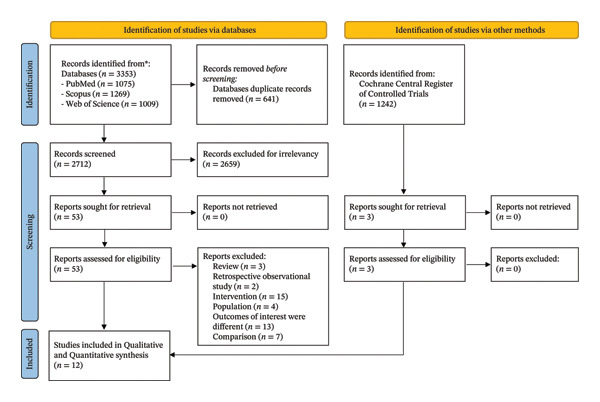
From: Page MJ, McKenzie JE, Bossuyt PM, Boutron I, Hoffmann TC, Mulrow CD, et al. The PRISMA 2020 statement: an updated guideline for reporting systematic reviews. BMJ 2021; 372:n71. https://doi:10.1136/bmj.n71. For more information, visit: https://www.prisma-statement.org/.

A total of 4595 records were identified in the initial research. Six hundred and forty‐one records were excluded as duplicates. A total of 2659 articles found through search databases and 1239 studies identified via the Cochrane registry were removed due to irrelevancy. Following a precise full‐text appraisal, 44 records were excluded due to inconsistency with our inclusion criteria. Eventually, 12 studies were entered in the final review (Figure 1).

Table 1 illustrates the main characteristics of selected studies. Ten studies were published between 2002 and 2009, and two studies were published from 2011 to 2023. All the studies had RCT design. These studies compared the effect of orlistat alone and in combination with placebo or other lipid‐lowering medications.

**TABLE 1:** Study characteristics.

Author	Year	Design	Blinding	Follow‐up duration	Intervention	N	Age (years) (M±SD)	Female (%, n)	BMI(kg/m2) baseline	Waist circumference (cm) baseline	Waist‐to‐height ratio baseline	Total cholesterol (mg/dL) baseline	LDL cholesterol(mg/dL) baseline	HDL cholesterol(mg/dL) baseline	Triglyceride(mg/dL) baseline	Serum glucose baseline	Apolipoprotein b baseline
Feng et al.	2023	RCT	single‐blind	24 weeks	control group	39	44 ± 12	43.60	28.3 ± 2.2	93.9 ± 8.1	0.9 ± 0.04	197 ± 39	124 ± 31	42 ± 8	93 ± 108	93.6 ± 21.6	100 ± 20
					orlistat group	32	46 ± 11	12.50	29.8 ± 2.7	95.6 ± 8.0	0.9 ± 0.1	193 ± 50	120 ± 31	46 ± 12	66 ± 66	99 ± 25.2	90 ± 20
					experimental diet group	32	39 ± 12	37.50	28.8 ± 3.2	93.9 ± 8.2	0.7 ± 0.05	197 ± 31	120 ± 19	43 ± 8	73 ± 35	99 ± 19.8	80 ± 10

Kwon et al.	2021	RCT	double‐blind	12‐week clinical trial	placebo/phentermine	55	46.0 ± 11.3	78.6	*29.8*	102.8 ± 9.5	0.95 ± 0.01	206 ± 40	131 ± 30	52 ± 9	130 ± 16	100.8 ± 3.15	N/A
					orlistat/phentermine	57	45.5 ± 12.5	77.2	30.8	104.1 ± 9.5	0.98 ± 0.01	194 ± 35	119 ± 24	52 ± 13	100 ± 15	102.6 ± 4.95	N/A

Florentin et al.	2009	RCT	single‐blind	3 months	Ezetimibe + Rimobanat (20 mg/daily)	15	52.4 ± 15.5	60	32.4 ± 5.4	112.4 ± 12.8	N/A	271 ± 19	184 ± 27	56 ± 7	155 ± 67	98 ± 19	169.0 ± 46.8
					ezetimibe (10 mg/day) plus orlistat (120 mg, 3 times a day with meals)	15	54.7 ± 12.2	60	34.0 ± 4.4	110.8 ± 13.9	N/A	269 ± 50	181 ± 40	55 ± 8	167 ± 61	99 ± 9	162.3 ± 35.2

Nakou et al.	2008	RCT	single‐blind	6 months	group O, orlistat 120 mg 3 times a day (TID)	29	54 ± 9	75.80	35.7 ± 6.7	120 ± 14	N/A	247 ± 44	164 ± 38	52 ± 9	N/A	106 ± 16	112 ± 11
					group E, ezetimibe 10 mg/day	28	55 ± 11	71	35.8 ± 6.0	118 ± 18	N/A	251 ± 31	166 ± 27	54 ± 7	N/A	102 ± 16	115 ± 14
					group OE, orlistat 120 mg TID þ ezetimibe 10 mg/day	29	55 ± 10	68.90	35.5 ± 6.1	117 ± 15	N/A	256 ± 32	172 ± 32	53 ± 7	N/A	109 ± 21	117 ± 21

Chou et al.	2007	RCT	Not specified	36 weeks	sibutramin	20	49 ± 12	90		98.90 ± 15.79	N/A	199.7 ± 38.9	143.1 ± 34.5	32.7 ± 10.5	126.4 ± 66.5	195.7 ± 57.6	N/A
					orlistat	14		78.50		100.79 ± 11.71	N/A	200.0 ± 40.0	141 ± 31.0	34 ± 11.8	118.2 ± 69.6	195.8 ± 57.4	N/A

Filippatos et al.	2007	RCT	single‐blind	6 months	orlistat	28	52 ± 9	82	35 ± 6	116 ± 13	N/A	259 ± 48	4.3 ± 1.1(164 ± 42)	52 ± 10	216 ± 110.25	104 ± 14	113 ± 33
					fenofibrate	28	54 ± 11	71	34 ± 6	114 ± 12	N/A	257 ± 29	157 ± 26	51 ± 8	240 ± 87.75	99 ± 12	111 ± 27
					orlistat + fenofibrate	27	52 ± 10	85	35 ± 6	117 ± 11	N/A	266 ± 46	167 ± 46	52 ± 11	242 ± 99.75	106 ± 14	117 ± 28

Filippatos et al.	2005	RCT	single‐blind	3 months	orlistat	29	51 ± 9	82	35 ± 6	116 ± 13	N/A	255 ± 46	166 ± 36	52 ± 10	203 ± 110.25	105 ± 15	N/A
					fenofibrate	29	54 ± 12	68	34 ± 7	113 ± 12	N/A	252 ± 28	153 ± 25	50 ± 8	247 ± 87.75	103 ± 11	N/A
					orlistat + fenofibrate	*28*	53 ± 10	85	35 ± 5	118 ± 10	N/A	265 ± 45	168 ± 42	52 ± 11	238 ± 99.75	106 ± 14	N/A

Berne et al.	2005	RCT	double blind	1 year	orlistat	111	58.9 ± 9.1	45	32.6 ± 3.1	108.0 ± 9.0	N/A	213 ± 39	120 ± 39	50 ± 12	101 ± 54	201.6 ± 46.8	110 ± 20
					placebo	109	59.3 ± 8.5	46	32.9 ± 3	109.0 ± 9.3	N/A	209 ± 43	116 ± 31	46 ± 8	108 ± 97	196.2 ± 45	110 ± 20

Derosa et al.	2005	RCT	double‐blind	12 months	orlistat	55	50 ± 4	50.80	32.7 ± 1.8	104 ± 7	0.9 ± 0.2	192 ± 19	121 ± 14	47 ± 5	143 ± 37	N/A	N/A
					sibutramine	58	51 ± 5	51.70	33 ± 1.7	103 ± 5	0.88 ± 0.1	195 ± 21	125 ± 14	47 ± 6	141 ± 38	N/A	N/A

Derosa et al.	2003	RCT	double blind	6 months and 1 year	Orlistat	27	51.6 ± 8.3	51.9	32 ± 1.3	100.8 ± 5.3	N/A	260 ± 20	195 ± 20	43 ± 4	132 ± 32	N/A	N/A
					Placebo	23	52.4 ± 10.2	52.2	31.7 ± 1	102.3 ± 6.2	N/A	265 ± 24	194 ± 22	41 ± 3.5	128 ± 25	N/A	N/A

Derosa et al.	2002	RCT	single‐blind	6 months and 1 year	Orlistat	28	55 ± 10	50	33 ± 1.4	98 ± 6	N/A	254 ± 25	189 ± 22	42 ± 4	137 ± 24	NA	N/A
					Placebo	29	56 ± 10	50	33.2 ± 1.3	97 ± 5	N/A	257 ± 22	190 ± 23	41 ± 3	146 ± 29	N/A	N/A

Miles et al.	2002	RCT	double‐blind	52 weeks	Orlsitat	254	53.7 ± 0.4	48	35.2 ± 0.2	N/A	N/A	209 ± 22	N/A	N/A	249 ± 10	208.8 ± 3.6	N/A
					Placebo	249	52.5 ± 0.4	48	35.6 ± 0.3	N/A	N/A	209 ± 22	N/A	N/A	233 ± 8	199.8 ± 3.6	N/A

Abbreviation: N/A: Not available.

These 12 studies were reported between 2002 and 2021. The overall participants were 1369, with 682 in the treatment group and 687 in the control category.

All included trials considered a hypocaloric diet (dietary restriction and lifestyle counseling) for participants in both arms. In addition, six trials explicitly reported an exercise component alongside dietary restriction [22–27]. Four trials explicitly specified a low‐fat diet [25, 28–30]. One trial explicitly specified a low‐carbohydrate diet [22]. Also, inter‐reviewer agreement for categorical extraction items was substantial to almost perfect (exercise: *κ* = 0.667; low‐fat diet: *κ* = 0.824; low‐carb diet: *κ* = 1.000; overall *κ* = 0.809). Inter‐reviewer reliability for continuous data extraction was high (ICC(2,1) absolute agreement = 0.999857354, 95% CI [0.9998 to 0.9999], based on 544 paired extracted cells) (Supporting Information 3).

### 3.1. Risk of Bias Assessment

**FIGURE 2:**
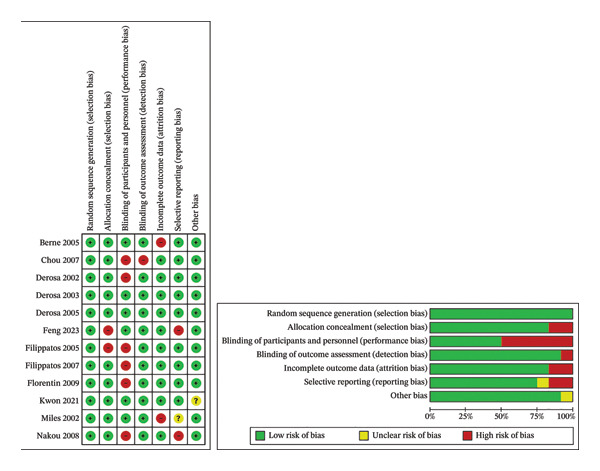
Risk of bias graph and summary.

The Cochrane study quality assessment tool was utilized (Figure 2). According to random sequence generation, as demonstrated in Figure 3, all 12 selected studies indicated an appropriate approach for randomization. In terms of allocation concealment, only two studies were found to have a bias [22, 25]; six of them blinded personnel and participants to avoid performance bias [22, 23, 26, 31–33]; outcome assessment was blinded in all the studies, except one [24]. Overall, three studies [23, 26, 33] achieved the highest quality, and four studies obtained the lowest rank in this evaluation [22, 24, 25, 29].

FIGURE 3Meta‐analysis of orlistat clinical effect for each outcome. (CI: confidence interval, SMD: standardized mean difference, SE: standard error).

**Figure:**
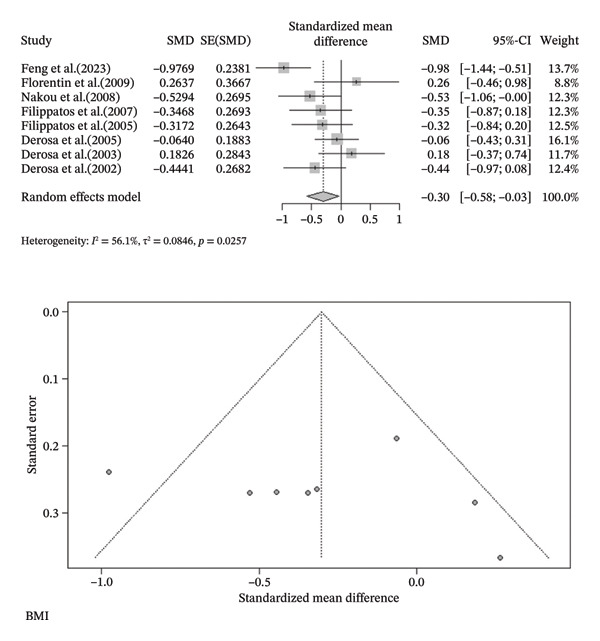
(a)

**Figure:**
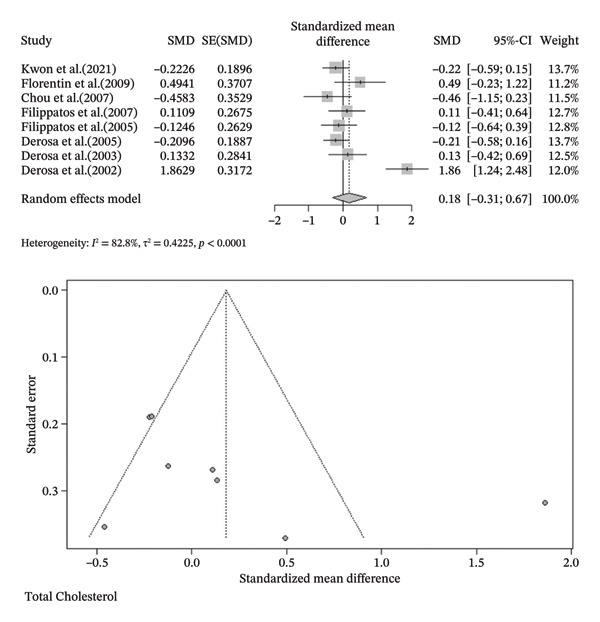
(b)

**Figure:**
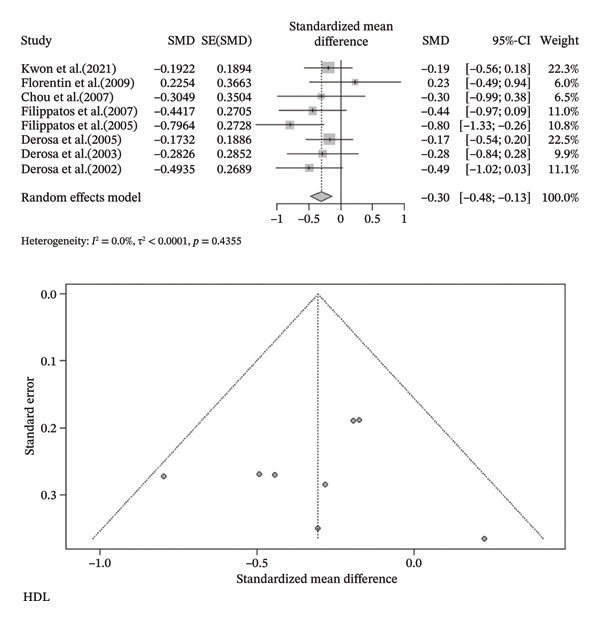
(c)

**Figure:**
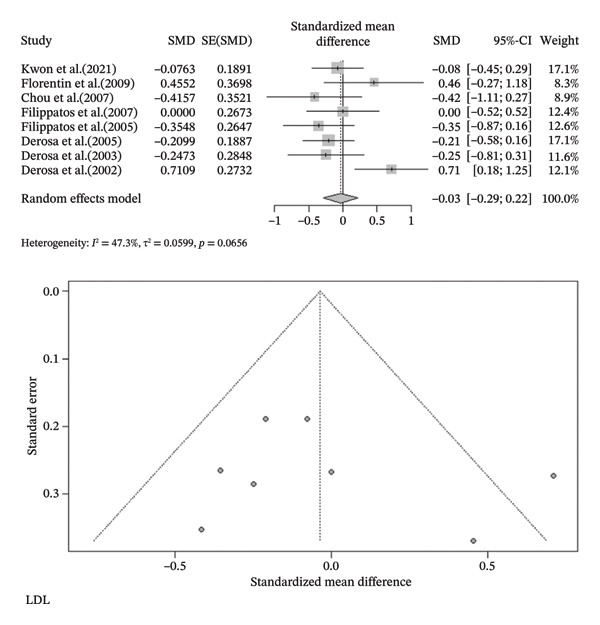
(d)

**Figure:**
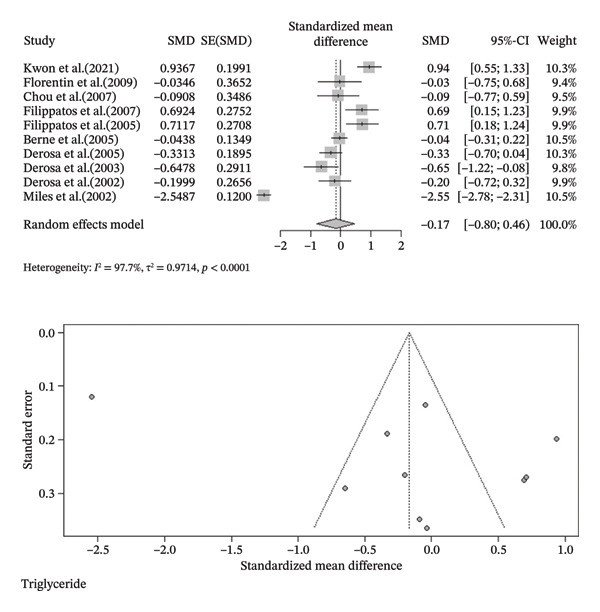
(e)

**Figure:**
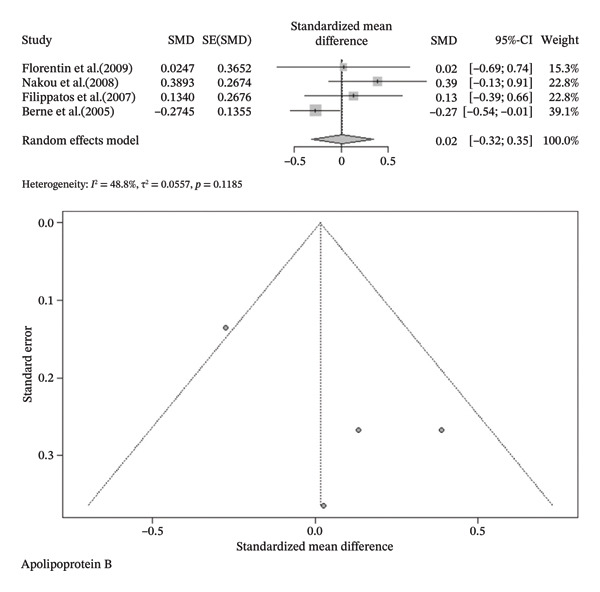
(f)

**Figure:**
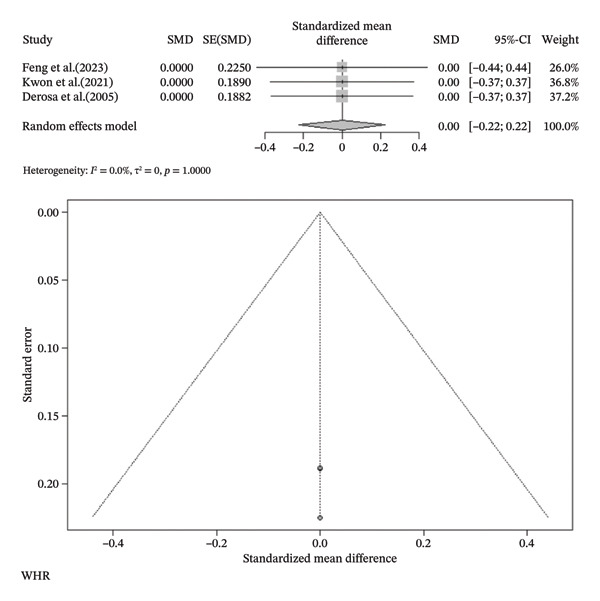
(g)

**Figure:**
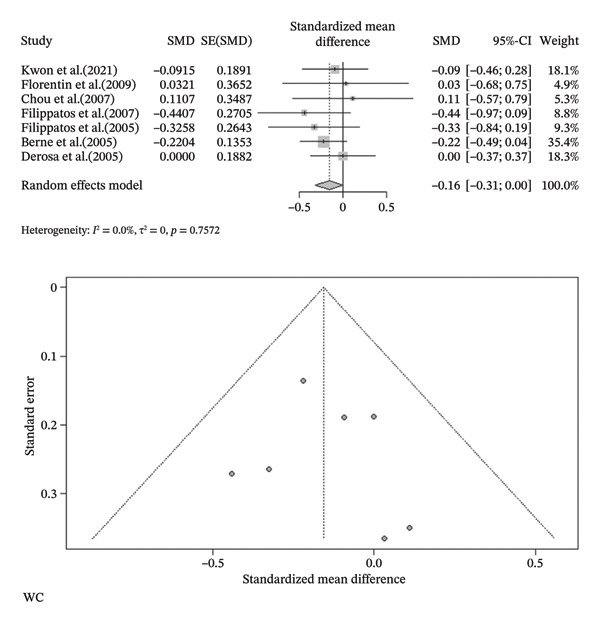
(h)

### 3.2. Orlistat Biomedical Results

Findings from the SMD for the changes in biomedical parameters are included in Table 2. Significant heterogeneity was found in TG and TC. Notably, heterogeneity in apolipoprotein B, BMI, and LDL‐C was moderate, and no heterogeneity was observed in HDL‐C, WC, and WHR. The Forest plot figure for all the variables was calculated, and Funnel plots were added to illustrate the publication bias of the studies. Calculations were performed based on the SMD size.

**TABLE 2:** Summary information of diagnostic variables and their comparisons with and without using orlistat.

Diagnostic parameter	Pooled effect size of difference			Heterogeneity				Imputed studies
	SMD [95% CI]	Z for SMD	*p*‐value	*I* ^2^	*τ* ^2^	D.F.	*p*‐value	
BMI	−0.3024 [‐0.5752; −0.0296]	−2.17	0.0298	56.1%	0.0846	7	0.0257	0
WC	−0.1562 [‐0.3138; 0.0015]	−1.94	0.0522	0.0%	0.0	6	0.7572	1
WHR	0.0000 [‐0.2249; 0.2249]	0.00	1.0000	0.0%	0.0	2	1.0000	0
HDL‐C	−0.3050 [‐0.4804; −0.1296]	−3.41	0.0007	0.0%	< 0.0001	7	0.4355	0
LDL‐C	−0.0349 [‐0.2853; 0.2154]	−0.27	0.7844	47.3%	0.0599	7	0.0656	0
Total Cholesterol	−0.0447 [‐0.4272; 0.3379]	−0.23	0.8190	84.0%	0.3176	9	< 0.0001	5
Triglyceride	−0.1668 [‐0.7979; 0.4642]	−0.52	0.6043	97.7%	0.9714	9	< 0.0001	4
Apolipoprotein B	0.0156 [‐0.3181; 0.3492]	0.09	0.9272	48.8%	0.0557	3	0.1185	2

As indicated in Table 2, orlistat leads to remarkable changes in BMI (SMD (%95CI): −0.3024 [‐0.5752; −0.0296], *I* ^2^ = 56.1%, *p*‐value (heterogeneity) = 0.0257), and HDL (SMD (%95CI): −0.3050 [‐0.4804; −0.1296], *I* ^2^ = 0.0%, *p*‐value (heterogeneity) = 0.4355). As a result, although orlistat has been found to be effective in reducing BMI, its impact on decreasing HDL‐C levels is not desirable. Furthermore, our findings found no statistically significant difference between the two groups in LDL‐C, WHR, apolipoprotein B, TG, WC, and TC after orlistat administration in the primary analysis (Table 2). Given signs of possible publication bias for several outcomes, we additionally report trim‐and‐fill adjusted estimates as a sensitivity analysis (Table 3), where WC and TG became statistically significant. Figure 3 indicates the Forest and Funnel plots for each parameter.

**TABLE 3:** Summary adjusted values and information of diagnostic variables following publication bias analysis.

Diagnostic parameter	Corrected pooled effect size of difference			Heterogeneity			
	SMD [95% CI]	Z for SMD	*p*‐value	*I* ^2^	*τ* ^2^	D.F.	*p*‐value
WC	−0.1712 [‐0.3248; −0.0176]	−2.18	0.0290	0.0%	0.0	7	0.7696
Total Cholesterol	−0.4413 [‐0.8847; 0.0021]	−1.95	0.0511	88.8%	0.6974	14	< 0.0001
Triglyceride	−0.8900 [‐1.6619; −0.1181]	−2.26	0.0238	97.9%	2.1014	13	< 0.0001
Apolipoprotein B	−0.2125 [‐0.5840; 0.1590]	−1.12	0.2622	67.7%	0.1476	5	0.0085

### 3.3. Subgroup Analysis and Meta‐Regression

**FIGURE 4:**
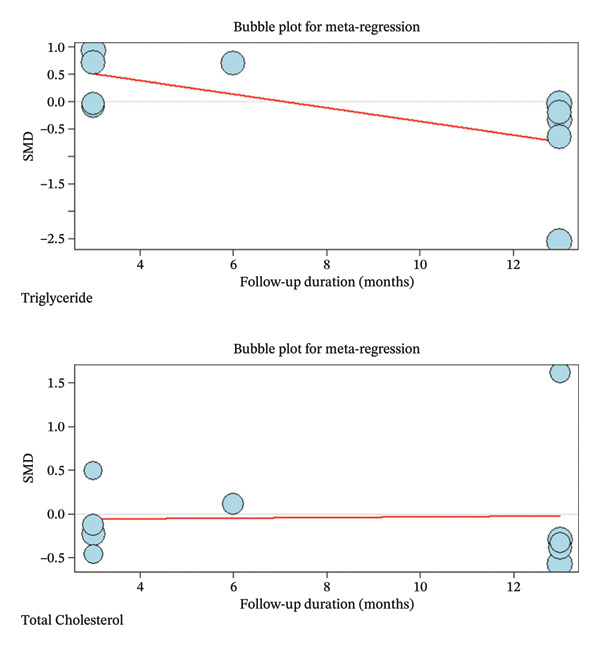
Meta‐regression bubble plots demonstrating the correlation between follow‐up duration and standardized mean changes.

Due to the high heterogeneity observed in TC and TG, subgroup analysis as primary and meta‐regression as secondary analysis for determining the association of orlistat treatment duration with TC and TG levels were performed. No meaningful changes were found in TG levels before and after 6 months following orlistat treatment (SMD (%95CI): 0.32 [‐0.05; 0.69], *I* ^2^ = 72.6%, *p*‐value (heterogeneity) = 0.0013, SMD (%95CI): −0.7635 [‐1.6843; 0.1573], *I* ^2^ = 98.3%, *p*‐value (heterogeneity) < 0.0001, respectively) and in TC quantities (SMD (%95CI): 0.18 [‐0.31; 0.67], *I* ^2^ = 82.8%, *p*‐value (heterogeneity) < 0.0001, SMD (%95CI): 0.29 [‐0.98; 1.57], *I* ^2^ = 94.0%, *p*‐value (heterogeneity) = < 0.0001, respectively) (Figure Supplemental materials). The high heterogeneity in TG and TC after 6 months of treatment suggests that TC and TG reduction varies with treatment duration. In a secondary analysis, meta‐regression showed that follow‐up duration accounted for 30% of the heterogeneity (*R* ^2^ = 30%) in TG. The analysis suggested a small but statistically significant decline in the TG‐lowering effect of orlistat over time (slope: −0.1239; 95% CI: −0.2355, −0.0123; *p*‐value = 0.0295; Z‐value = −2.1763). Furthermore, similar to subgroup analysis, meta‐regression results revealed no meaningful association between follow‐up duration and TC reduction (slope: 0.0036; 95% CI: −0.0823, 0.0894; *p*‐value = 0.9348; Z‐value = 0.0818). Besides, no residual heterogeneity was identified in TC following the treatment duration (*R* ^2^ = 0.00%) (Figure 4).

### 3.4. Subgroup Analysis of Combination Therapy vs Orlistat Monotherapy

In an exploratory analysis of trials that directly compared orlistat combined with lipid‐lowering therapy (e.g., statins/fibrates/ezetimibe) versus orlistat alone, no statistically significant differences were observed for anthropometric indices: BMI (SMD = −0.24, 95% CI −0.71 to 0.23; *I* ^2^ = 72.3%) and WC (SMD = 0.12, 95% CI −0.18 to 0.42; *I* ^2^ = 0.0%).

For lipid outcomes, combination therapy was associated with greater reductions in LDL‐C (SMD = −1.23, 95% CI −2.31 to −0.15; *I* ^2^ = 91.9%), TC (SMD = −1.52, 95% CI −2.45 to −0.60; *I* ^2^ = 88.6%), and TGs (SMD = −0.55, 95% CI −0.92 to −0.18; *I* ^2^ = 46.3%), compared with orlistat alone. This suggests a significant synergistic benefit when targeting both fat absorption and lipid synthesis simultaneously. The effect on HDL‐C favored combination therapy but did not reach conventional statistical significance (SMD = 0.50, 95% CI −0.03 to 1.02; *I* ^2^ = 73.1%) (Supporting Information 4).

### 3.5. Adverse Events

Multiple studies reported adverse events during orlistat administration [22, 23, 25, 29]. Gastrointestinal issues were the prevalent side effects demonstrated in patients when using orlistat in these studies. Distinctly, insomnia had the most occurrence rate in the Kwon et al. study [23]. Furthermore, steatorrhea as a gastrointestinal problem was found to be statistically meaningful in this study (*p*‐value = 0.02) [23]. Besides, no study discovered an elevation in AST and ALT levels or other liver enzymes.

### 3.6. Publication Bias Analysis

Visual analysis demonstrated asymmetry in Funnel plots, presenting possible publication bias in outcomes. In particular, the trim‐and‐fill method indicated 1, 5, 4, and 2 missing studies following WC, TC, TG, and apolipoprotein B analyses, respectively. Furthermore, the statistical significance of TC and apolipoprotein B remained consistent. However, in contrast to the initial findings, TG and WC were found to be statistically significant (SMD (%95CI): −0.8900 [−1.6619; −0.1181], *I* ^2^ = 97.9%, *p*‐value < 0.0001, SMD (%95CI): −0.1712 [−0.3248; −0.0176], *I* ^2^ = 0.0%, *p*‐value = 0.7696, respectively) (Table 3). While in the initial analysis, no statistical significance was discovered in TG and WC following orlistat use (Table 2). Besides, the trim‐and‐fill method was utilized in subgroup analysis, in particular TG levels before and after 6 months of orlistat treatment (SMD (%95 CI): 0.5228 [0.1452; 0.9004], *I* ^2^ = 81.1%, *p*‐value < 0.0001, SMD (%95 CI): −2.1061 [−3.5055; −0.7068], *I* ^2^ = 99.2%, *p*‐value < 0.0001, respectively), demonstrating an effective response of orlistat in a long‐term duration. Because these findings are sensitive to publication‐bias adjustment and the certainty of evidence for these outcomes was low/very low (Table 4), they should be interpreted cautiously.

**Table 4:** GRADE summary of findings.

Outcome	Number of studies	Design	Number of patients		SMD [95% CI]	Risk of bias	Inconsistency	Indirectness	Imprecision	Publication bias	Certainty of evidence	Importance
			Control	Intervention								
BMI	8	RCT	249	251	−0.3024 [‐0.5752; −0.0296]	Very serious	Serious	Not serious	Serious	Undetected	⊕ΟΟΟ Very low	Critical
WC	7	RCT	314	309	−0.1562 [‐0.3138; 0.0015]	Very serious	Not serious	Not serious	Not serious	Strongly suspected	⊕⊕ΟΟ Low	Critical
WHR	3	RCT	152	152	0.0000 [‐0.2249; 0.2249]	Serious	Not serious	Not serious	Serious	Undetected	⊕⊕ΟΟ Low	Important
TG	10	RCT	620	613	−0.1668 [‐0.7979; 0.4642]	Very serious	Very serious	Not serious	Serious	Strongly suspected	⊕ΟΟΟ Very low	Critical
TC	10	RCT	620	613	−0.0447 [‐0.4272; 0.3379]	Very serious	Very serious	Not serious	Not serious	Strongly suspected	⊕ΟΟΟ Very low	Important
HDL‐C	8	RCT	257	253	−0.3050 [‐0.4804; −0.1296]	Very serious	Not serious	Not serious	Not serious	Undetected	⊕⊕ΟΟ Low	Critical
LDL‐C	8	RCT	257	253	−0.0349 [‐0.2853; 0.2154]	Very serious	Serious	Not serious	Not serious	Undetected	⊕⊕ΟΟ Low	Important
Apolipoprotein B	4	RCT	180	183	0.0156 [‐0.3181; 0.3492]	Very serious	Serious	Not serious	Serious	Strongly suspected	⊕ΟΟΟ Very low	Important

### 3.7. Certainty of the Evidence

Table 4 presents the overall certainty of evidence for each outcome. The overall certainty of evidence was low for HDL‐C, LDL‐C, WC, and WHR and was very low for BMI, TG, TC, and apolipoprotein B. Both LDL‐C and HDL‐C were downgraded due to a very serious risk of bias. Also, BMI was downgraded to very low‐quality evidence, suggesting a small reduction compared to the control group due to a very serious risk of bias and serious inconsistency and imprecision. However, no publication bias was detected in BMI. TG and TC were downgraded owing to significant concerns regarding risk of bias, inconsistency, and potential publication bias.

## 4. Discussion

Our study unveiled a remarkable decline in HDL‐C and BMI following orlistat administration in the intervention group in comparison with the control group. Likewise, including ApoB and WHR adds clinical specificity beyond LDL‐C and BMI, because ApoB reflects atherogenic particle burden and WHR better captures central adiposity or visceral fat distribution. While the reduction in BMI suggests a favorable effect, the associated decline in HDL‐C may be undesirable, given its protective role in cardiovascular health. On the other hand, no noteworthy change was observed in other parameters of the study. Our findings suggest that as follow‐up duration increases, the TG‐lowering effect of orlistat decreases. However, the decline in effect over time is modest. Various studies have found similar results [34–36]. Gabriel et al. found that postprandial orlistat can significantly prevent TG from increasing and minimize the risk of atherosclerosis. Their study population consisted of 31 adults, who were fed half‐percent fat meals and took 120 mg after each meal. Similarly, they did not observe meaningful changes in HDL, LDL, and TC parameters. Also, they discovered that very low‐density lipoprotein (VLDL) was another clinical parameters that underwent a meaningful change following orlistat use as well as TG [35]. Besides, Wierzbicki et al. in England discovered that a combination of orlistat with fibrate‐statin drugs can significantly accelerate treatment in patients with severe hypertriglyceridemia [37]. In contrast, some of the studies demonstrated different results, unusefulness of orlistat in TG level [38–40]. For instance, Esmail et al. discovered that the orlistat effect on TG and TC was nonsignificant, despite its beneficial role in improving plasma free fatty acids (FFAs) and TG‐glucose index (TyG‐index) [41]. Possible reasons can be reflected in the short follow‐up of 12 weeks, lower‐than‐standard dosing regimen (120 mg/daily, instead of 360 mg/daily or 120 mg T.I.D), and high TG variability (SD = 84.4 mg/dL). Besides, this study included substantial metabolic comorbidity (e.g., diabetes and thyroid disorders are reported). In NAFLD/insulin resistance, hepatic VLDL‐TG production and de novo lipogenesis can remain high, potentially blunting fasting TG changes even if other markers like FFAs and TyG index improve. To explore whether baseline lipid phenotype explains heterogeneity in TG outcomes, we conducted subgroup analyses stratified by study mean baseline TG (< 150 vs ≥ 150 mg/dL). Neither subgroup demonstrated a statistically significant pooled TG effect, and the ≥ 150 mg/dL subgroup was highly imprecise with substantial heterogeneity due to the small number of trials and an influential study. Overall, in this exploratory, study‐level analysis baseline TG alone does not account for between‐study variability, and other factors (e.g., follow‐up duration, dose, population characteristics) may be more important effect modifiers. In addition to baseline lipid phenotype and follow‐up duration, variability in lifestyle cointerventions may have contributed to inconsistent TG findings across trials. Because orlistat’s mechanism depends on dietary fat intake, differences in the strictness of low‐fat hypocaloric prescriptions and adherence monitoring may have reduced between‐group contrasts and partially explain inconsistent TG effects across trials.

In our meta‐analysis, the positive effect of orlistat on BMI was found. Identically, in 2019, a study performed by Gorgojo‐Martínez et al. revealed the beneficial impact of orlistat on weight loss [42]. However, they identified the superiority of liraglutide to orlistat in reducing body weight within a follow‐up duration of 7 months. In parallel, Feng et al. discovered that orlistat’s effects on BMI surpass the high‐protein/lower‐carbohydrate diet [22]. Notably, Gorgojo‐Martínez et al. found that orlistat plays a pivotal role in enhancing vascular endothelial cell activities and flow‐mediated dilation regardless of weight loss, which is in line with results attained from several studies investigating obese patients [43–45]. In a similar study conducted in Malaysia, patients who received 120 mg of orlistat three times a day demonstrated improvements in microvascular endothelial function and arterial stiffness, as well as reductions in blood pressure and heart rate. These effects were not observed in patients who were administered 10 mg of sibutramine daily. A meta‐analysis performed by Sahebkar et al. identified that orlistat treatment can drastically decrease systolic blood pressure (SBP) by a weighted mean difference (WMD) of 1.29 mmHg and diastolic blood pressure (DBP) by a WMD of 1.23 mmHg and can also potentially contribute to lowering fasting plasma glucose [11, 15]. For instance, an observational retrospective study found that orlistat can lead to a 14% decrease in plasma glucose following a 24‐week treatment span [46]. An avalanche of studies has confirmed these findings [23, 33, 42, 46, 47].

Furthermore, our meta‐regression suggested that the magnitude of TG reduction attenuated with longer follow‐up (slope = −0.1239), implying a time‐dependent waning of effect. Several plausible mechanisms may explain this pattern. First, long‐term adherence to orlistat and to the concomitant lifestyle program (hypocaloric diet and, in some trials, structured exercise and/or macronutrient targets) may decline over time, and treatment discontinuation is common in multiyear trials (e.g., only 52% of participants in the orlistat arm completed the 4‐year XENDOS study) [48]. Second, gastrointestinal adverse effects and dietary fat intake strongly influence tolerability; gastrointestinal‐related events are often most frequent early and decreased over time in the XENDOS study, which may reflect dietary adaptation or discontinuation or dose omission among less tolerant participants [48, 49]. Third, compensatory shifts in dietary composition over longer follow‐up could blunt TG improvements; for example, low‐fat diets that are relatively higher in carbohydrate (depending on what replaces fat) can increase plasma TG partly via reduced VLDL‐TG clearance [50]. Finally, emerging evidence indicates that orlistat alters gut microbial composition and gut‐derived metabolites, which could modulate lipid handling over time, although direct longitudinal clinical data remain limited [51].

Remarkably, a meta‐analysis performed on nonalcoholic fatty liver disease (NAFLD) individuals discerned that orlistat cannot improve liver fibrosis scores. As a result, alternative drugs should be considered as the first‐choice administration for NAFLD patients [52]. Although, treatment with orlistat was associated with significant reductions in serum alanine aminotransferase (ALT) and aspartate aminotransferase (AST) levels, suggesting potential biochemical improvement despite the lack of histological benefit [52]. In contrast, another meta‐analysis conducted by Zahmatkesh et al. demonstrated that orlistat can potentially improve NAFLD patients and their related metabolic inducing factors for a 12‐week duration. This discrepancy can be owing to not measuring the liver fibrosis score [53]. The results were achieved based on ALT and AST analyses. Furthermore, they found that orlistat cannot statistically affect the TG level, which is against our findings [53]. Notably, in a different finding in the NAFLD population, orlistat was found to have productive effects on NAFLD and its metabolic factors, including TG. Also, they found that orlistat can have slight positive effects on liver fibrosis scores [54].

We found through various studies that orlistat does not influence HDL‐C and LDL‐C levels. This hypothesis has roots in the origin and formation of LDL‐C and HDL‐C in our bodies. Thirty percent of the entire cholesterol in the body originates from the diet. Meanwhile, the majority of the body’s cholesterol, about 70%, is synthesized in the body’s tissues, mainly in the liver [55]. Reasonably, major lipid‐forming LDL‐C and HDL‐C particles are cholesterol [56]. Consequently, although orlistat reduces cholesterol absorption from daily meals, its impact on TCs remains minimal. This is because most cholesterol in the body is endogenously produced, primarily in the liver. Ultimately, LDL and HDL cholesterol levels do not undergo a noticeable change during orlistat consumption. Studies conducted so far have stated that orlistat exerts a mild influence on TC, lowering its levels [23, 31].

Besides, our analysis unveiled meaningful changes in WC, following orlistat administration. These findings align the most recent meta‐analysis study performed in 2023 [57]. They found that orlistat is effective in attenuating WC [57]. However, they did not find any significant impacts of orlistat on BMI and body weight, which is against our findings. Also, no meaningful impact on WHR was identified. As WHR is directly calculated through the WC and hip circumference (HC) parameters, it is reasonable that no changes in WHR can be attributed positive impact of orlistat on WC and HC variables.

Apolipoprotein B, a structural protein in lipoproteins, including LDL and VLDL, plays a pivotal role in indicating cardiovascular risk levels more accurately than LDL‐C [58]. We did not find a notable change in apolipoprotein B following orlistat use. Since orlistat is a lipase inhibitor, and apolipoprotein B is encoded through the APOB gene, it is logical that apolipoprotein B synthesis is independent of orlistat consumption [58, 59].

Combination therapy may partly explain divergent lipid findings across trials. In our exploratory head‐to‐head analysis, trials using orlistat plus a lipid‐lowering agent showed larger reductions in LDL‐C, TC, and TGs compared with orlistat alone, whereas BMI and WC were not meaningfully different between regimens. This pattern is biologically plausible because statins/ezetimibe primarily lower LDL‐C and TC and fibrates primarily lower TG, while orlistat’s principal contribution is reduced fat absorption and weight loss. However, these pooled estimates had substantial heterogeneity (particularly for LDL‐C and TC) and were derived from a limited number of studies with different drug classes and populations. Thus, the findings should be considered hypothesis‐generating rather than definitive evidence of superiority.

Our GRADE assessment revealed that the overall quality of evidence supporting the effects of interventions on anthropometric and lipid‐related outcomes ranged from very low to low. The evidence for BMI and WC, two key markers of obesity and metabolic health, was particularly weak due to high risk of bias in the included studies. Notably, the evidence for HDL‐C and LDL‐C was of low quality, but no publication bias was detected for them. This suggests that while the results should be interpreted cautiously, they may be more reliable compared to other lipid markers. The effect sizes were adjusted for variables, as publication bias was found to enhance the accuracy and precision of our findings.

One limitation of our study is the variability in the patient populations across the studies included in the meta‐analysis. Differences in baseline lipid levels, underlying comorbidities, and concurrent medications may account for some of the observed heterogeneity. Additionally, the absence of studies investigating WHR and fat‐free mass, critical markers for atherosclerotic risk, has led to an inability to accurately evaluate the effect of orlistat on WHR, fat mass, and fat‐free mass indicators. Further studies with larger sample sizes and longer durations, examining the long‐term effects of orlistat, particularly in combination with other lipid‐lowering agents, are warranted to better understand its role in reducing overall cardiovascular risk.

## 5. Conclusion

In conclusion, our findings suggest that orlistat plays a pivotal role in changing BMI and HDL‐C levels. Although its effect on HDL‐C is not beneficial due to HDL‐C’s protective role in cardiovascular health, in the primary meta‐analysis, reductions in TG and WC did not reach statistical significance, while trim‐and‐fill–adjusted estimates suggested potential decreases in TG and WC, demonstrating that these results are sensitive to possible publication bias. Although trim‐and‐fill–adjusted analyses suggested a potential TG reduction in longer follow‐up, the certainty of evidence is very low and heterogeneity was extreme; therefore, the presence and magnitude of any long‐term TG benefit remain uncertain. No significant impact on TG was observed following short‐term orlistat treatment. However, a modest positive effect may become apparent with long‐term use. While the effects on other parameters such as TC and LDL‐C remain without change, orlistat may still provide benefits for patients with obesity. Future research focusing on long‐term outcomes and combination therapies will help clarify the optimal role of orlistat in cardiovascular risk management.

## Author Contributions

Alireza Khodadadiyan and Hamed Bazrafshan drissi: conceptualization. Kimiya Kolaei, Parmida Aminzadeh, Yalda Khazraei, Maryam Feili, and Maliheh Kamali: data curation. Alireza Khodadadiyan: formal analysis. Hamed Bazrafshan drissi, Alireza Arzhangzadeh, Mehrasa Hosseini, Golnaz Yazdanpanah, Maliheh Kamali, Alireza Khodadadiyan, Maryam Feili, Yalda Khazraei, Parmida Aminzadeh, and Kimiya Kolaei: methodology. Alireza Khodadadiyan and Hamed Bazrafshan drissi: investigation. Hamed Bazrafshan drissi: project administration. Mehdi Bazrafshan: resources. Hamed Bazrafshan drissi: supervision. Alireza Khodadadiyan: software. Alireza Khodadadiyan, Ali Shams, Yalda Khazraei, Mehdi Bazrafshan, Alireza Arzhangzadeh, Mehrasa Hosseini, Golnaz Yazdanpanah, Parmida Aminzadeh, and Maliheh Kamali: writing–original draft. Alireza Arzhangzadeh, Hamed Bazrafshan drissi, Kimiya Kolaei, Maliheh Kamali, Maryam Feili, Melika Ghaffari: writing–review and editing.

## Funding

No any kind of financial support was received for this study.

## Ethics Statement

The authors have nothing to report.

## Consent

The authors have nothing to report.

## Conflicts of Interest

The authors declare no conflicts of interest.

## Supporting Information

Additional supporting information can be found online in the Supporting Information section.

## Supporting information


**Supporting Information 1** Search strategy.

**Supporting Information 2** Supporting Information 2: Subgroup analysis.

**Supporting Information 3** Supporting Information 3: Intraclass correlation coefficient (ICC).

**Supporting Information 4** Supporting Information 4: Orlistat combination therapy analysis.

## Data Availability

The data that support the findings of this study are available from the corresponding author upon reasonable request.
