# Supplementary material for: Long‐Term Effects of Orlistat on Lipid Metabolism and Anthropometric Indices: A Meta‐Analysis of Clinical Trials
Source: J Obes. 2026 Feb 23;2026:9068305. doi: 10.1155/jobe/9068305 (PMC12927897; doi:10.1155/jobe/9068305)
Supplement: Supplementary file 1 — Supporting Information 1 Search strategy. [file JOBE-2026-9068305-s001.docx]

Pubmed

**((Cardiovascular Diseases[MeSH Terms]) OR (cardiovascular system[MeSH Terms]) OR (Cardiovascular disease[Title/Abstract]) OR (Atherosclerosis[Title/Abstract]) OR (ischemic heart disease[Title/Abstract])) OR ((HDL-C[Title/Abstract]) OR (HDL-cholesterol[Title/Abstract]) OR (LDL-C[Title/Abstract]) OR (LDL-cholesterol[Title/Abstract]) OR (body weight[Title/Abstract]) OR (Cholesterol[MeSH Terms]) OR (hyperlipidemia[Title/Abstract]) OR (hyperlipidaemia[Title/Abstract]) OR (hyperlipidemic[Title/Abstract]) OR (hyperlipidaemic[Title/Abstract]) OR (dyslipidemia[Title/Abstract]) OR (dyslipidaemia[Title/Abstract]) OR (dyslipidemic[Title/Abstract]) OR (dyslipidaemic[Title/Abstract]) OR (triglycerides[Title/Abstract])) AND (Risk Factors[MeSH Terms]) AND ((Orlistat[Title/Abstract]) OR (Orlistat[MeSH Terms]) OR (lipid lowering drugs[Title/Abstract]) OR (lipid lowering medications[Title/Abstract]))**

1075 by date of 1/19/2025

Scopus

(TITLE-ABS-KEY (Orlistat OR lipid lowering drugs OR lipid lowering medications)) AND (TITLE-ABS-KEY ("Risk Factors")) AND (TITLE-ABS-KEY ("cardiovascular disease" OR "cardiovascular system" OR "Cardiovascular Disease" OR Atherosclerosis OR "ischemic heart disease")) AND (TITLE-ABS-KEY (HDL-C OR HDL-cholesterol OR LDL-C OR LDL-cholesterol OR "body weight" OR diabetes OR hyperlipidemia OR hyperlipidaemia OR hyperlipidemic OR hyperlipidaemic OR dyslipidemia OR dyslipidaemia OR dyslipidemic OR dyslipidaemic OR cholesterol OR triglycerides))

1269 by date of 1/19/2025

Web of science

TS = (((orlistat) OR (lipid-lowering medication)) AND (risk factor) AND ((cardiovascular) OR (CVD) OR (IHD) OR (Atherosclerosis) OR (Ischemic Heart Disease)) AND ((HDL-C) OR (hyperlipidemia) OR (hyperlipidaemia) OR (hyperlipidemic) OR (hyperlipidaemic) OR (dyslipidemia) OR (dyslipidaemia) OR (dyslipidemic) OR (dyslipidaemic) OR (cholesterol) OR (triglycerides) OR (HDL-cholesterol) OR (LDL-C) OR (LDL-cholesterol)))

1009 by date of 1/19/2025

cardiovascular OR CAD OR IHD OR atherosclerosis OR Ischemic Heart Disease OR coronary artery disease in Title Abstract Keyword AND risk-factor OR risk factor in Title Abstract Keyword AND orlistat OR lipid lowering medicaiton OR lipid lowering drugs in Title Abstract Keyword AND HDL-C OR HDL-cholesterol OR LDL-C OR LDL-cholesterol OR body weight OR HDL OR LDL OR low density lipoprotein OR high density lipoprotein OR hyperlipidemia OR hyperlipidaemia OR hyperlipidemic OR hyperlipidaemic OR dyslipidemia OR dyslipidaemia OR dyslipidemic OR dyslipidaemic in Title Abstract Keyword - (Word variations have been searched)

1242 by date of 1/19/2025

Total references = 4595

Duplicates = 641
