# Supplementary material for: Long‐Term Effects of Orlistat on Lipid Metabolism and Anthropometric Indices: A Meta‐Analysis of Clinical Trials
Source: J Obes. 2026 Feb 23;2026:9068305. doi: 10.1155/jobe/9068305 (PMC12927897; doi:10.1155/jobe/9068305)
Supplement: Supplementary file 2 — Supporting Information 2 Supporting Information 2: Subgroup analysis. [file JOBE-2026-9068305-s002.docx]

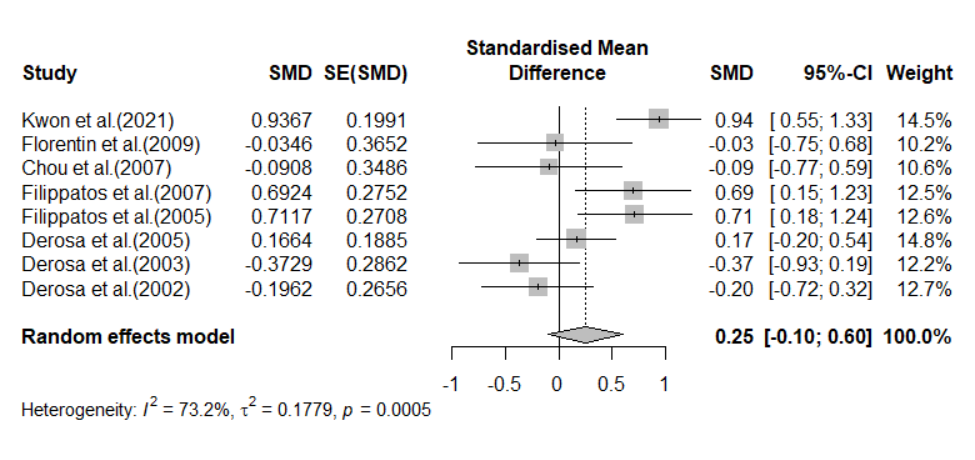

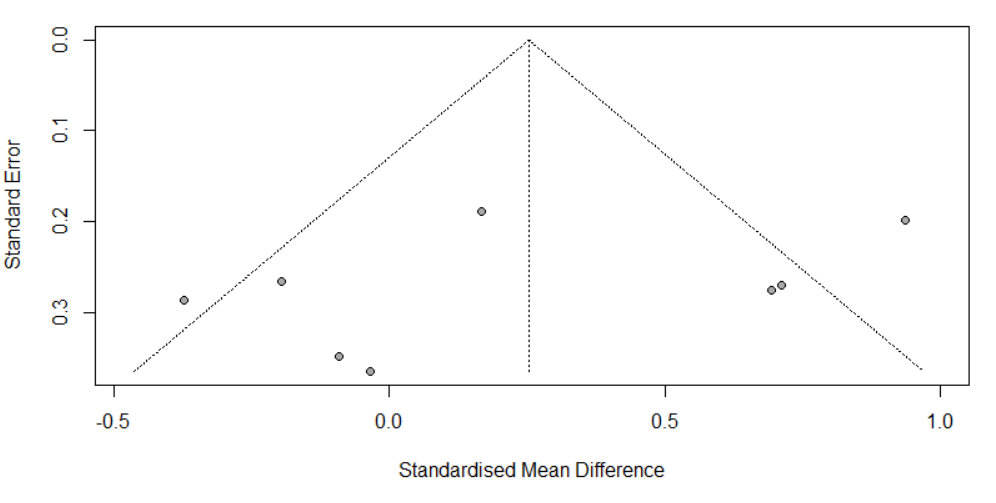


**Triglyceride (TG): Treatment duration<6 months**

**
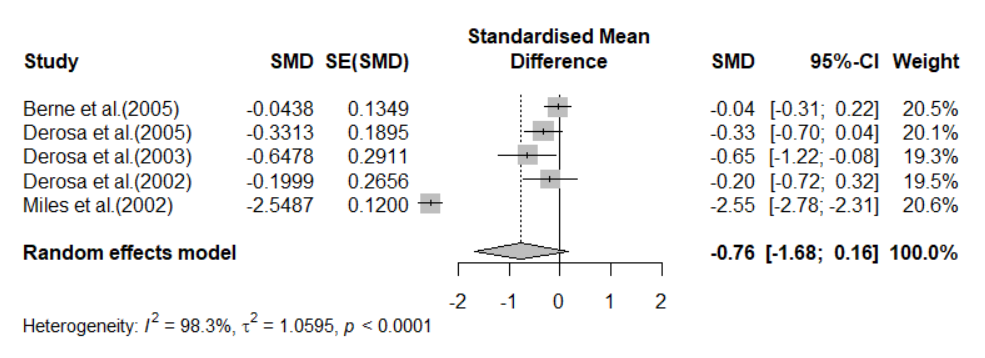
**

**
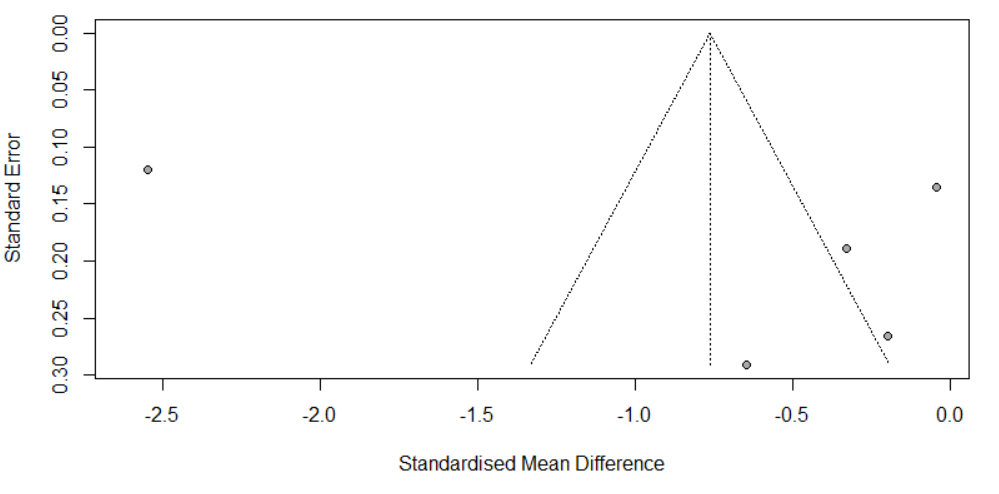
**

**Triglyceride (TG): Treatment duration>6 months**


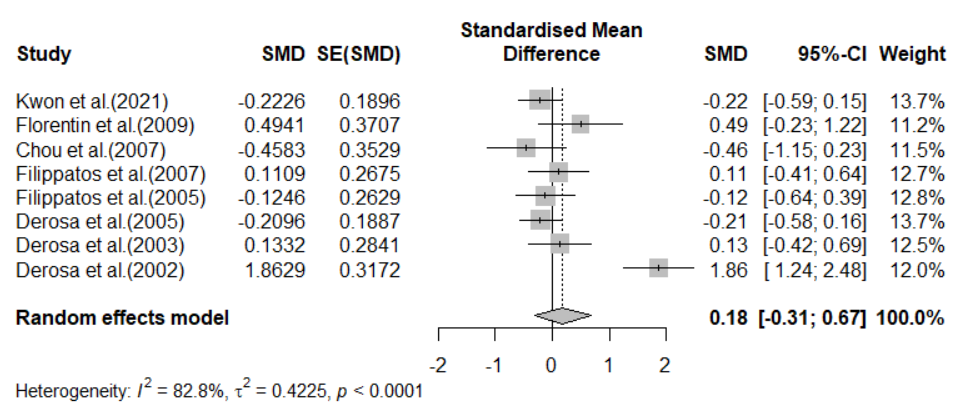


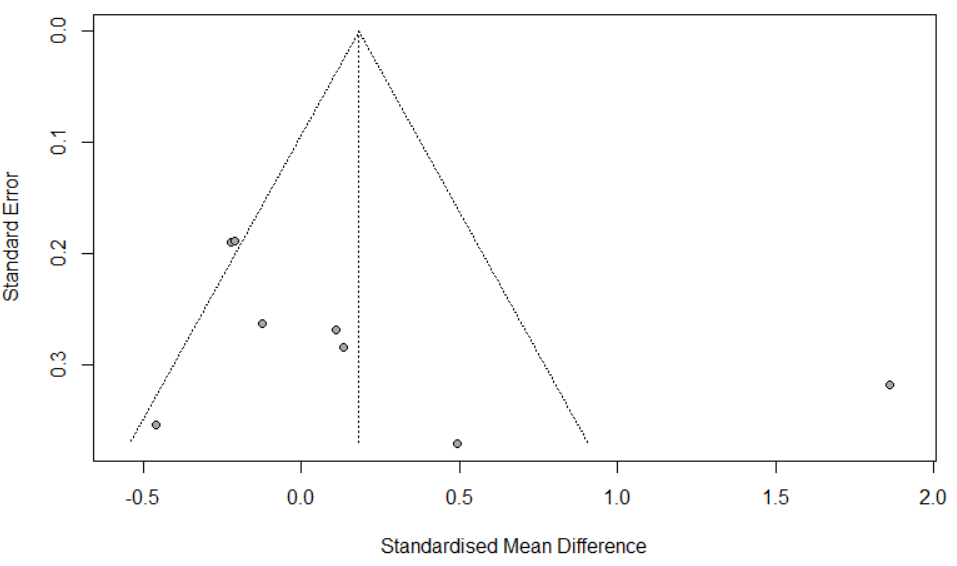


**TC: Treatment duration<6 months**

**
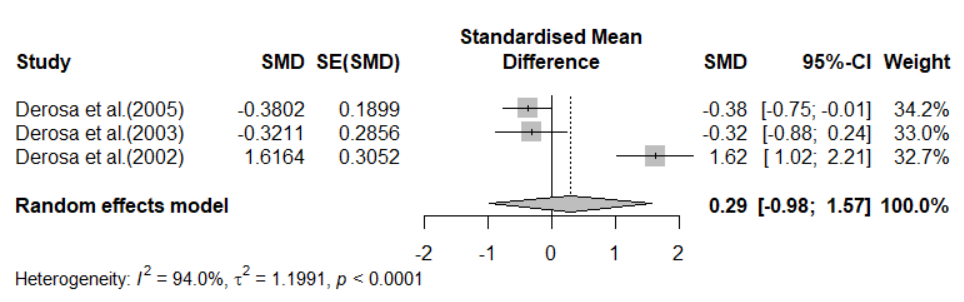

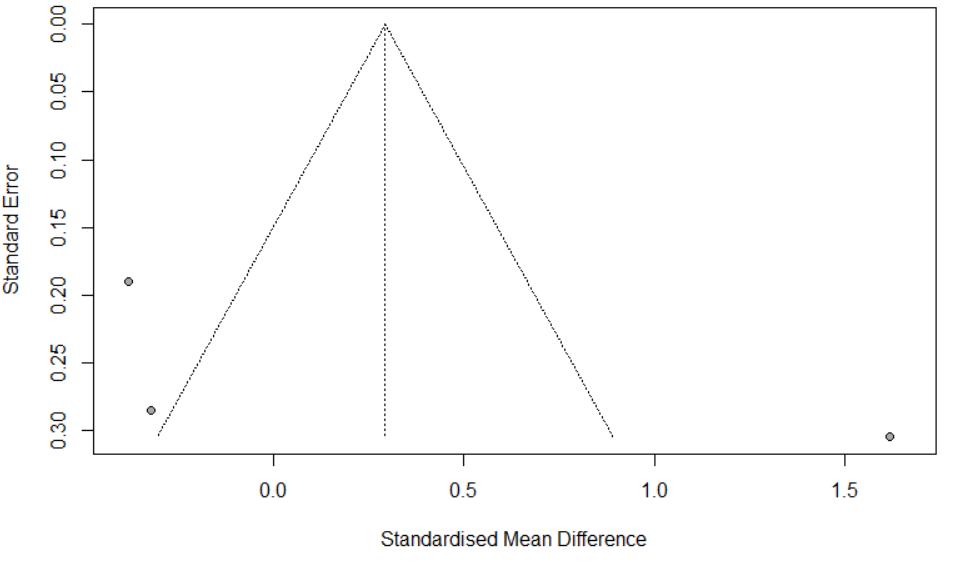
**

**TC: Treatment duration>6 months**

**
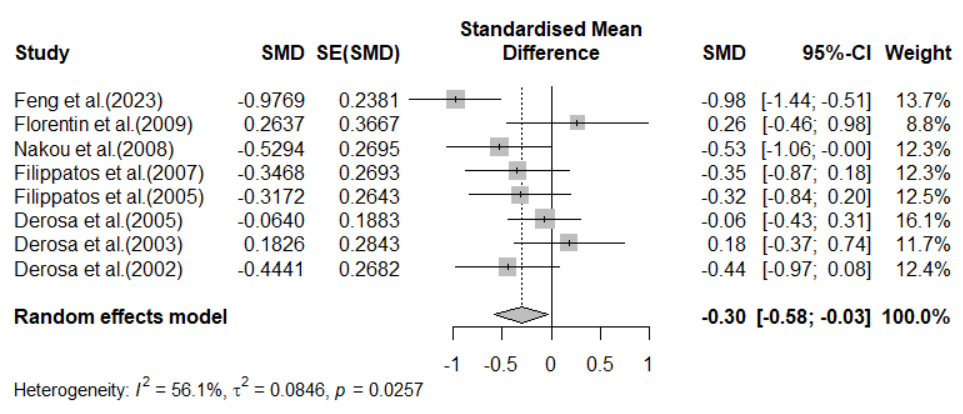
** **
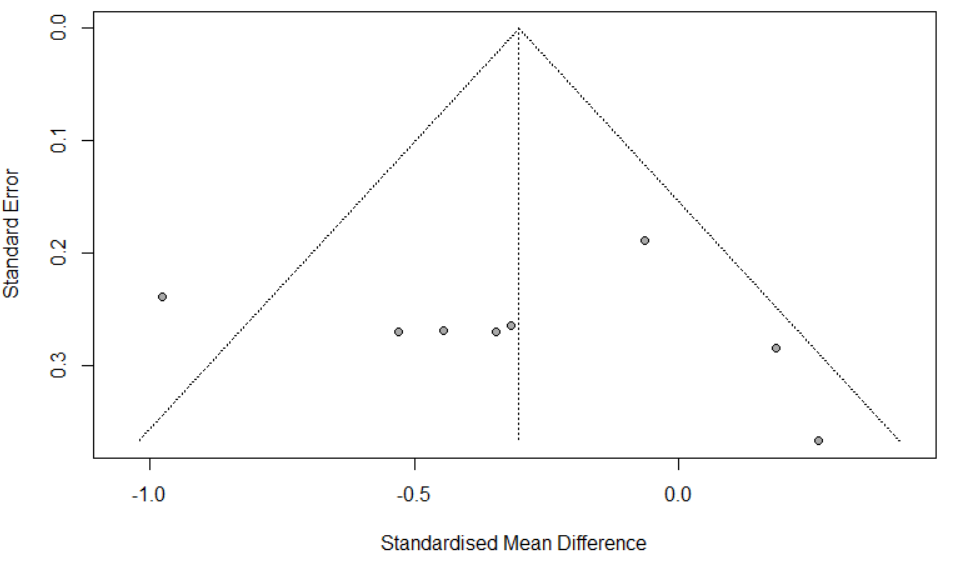
**

**BMI: Treatment duration>6 months**

**
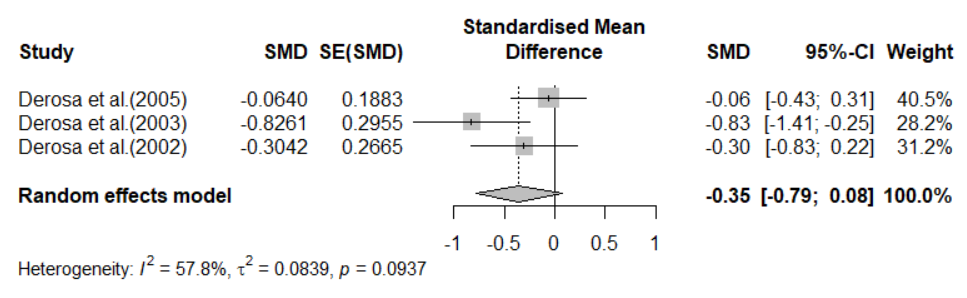
**

**
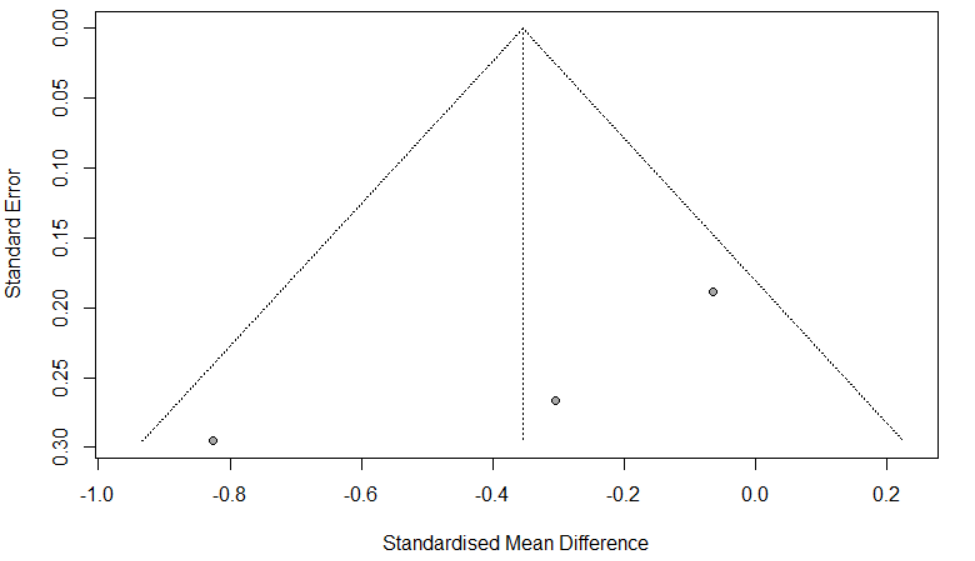
**

**BMI: Treatment duration>6 months**

**
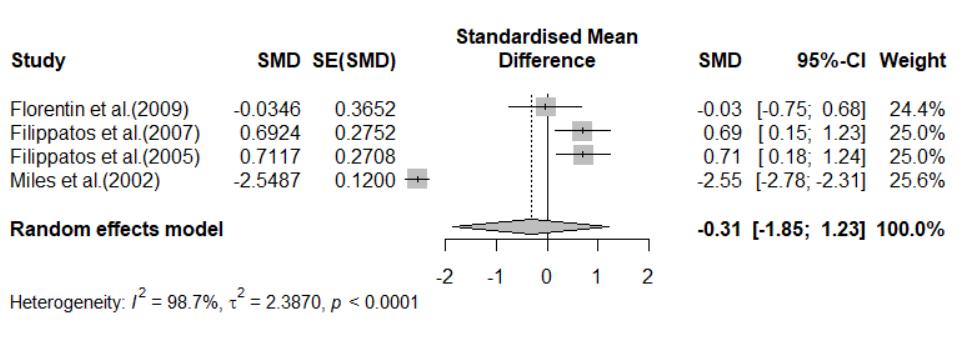
**

**
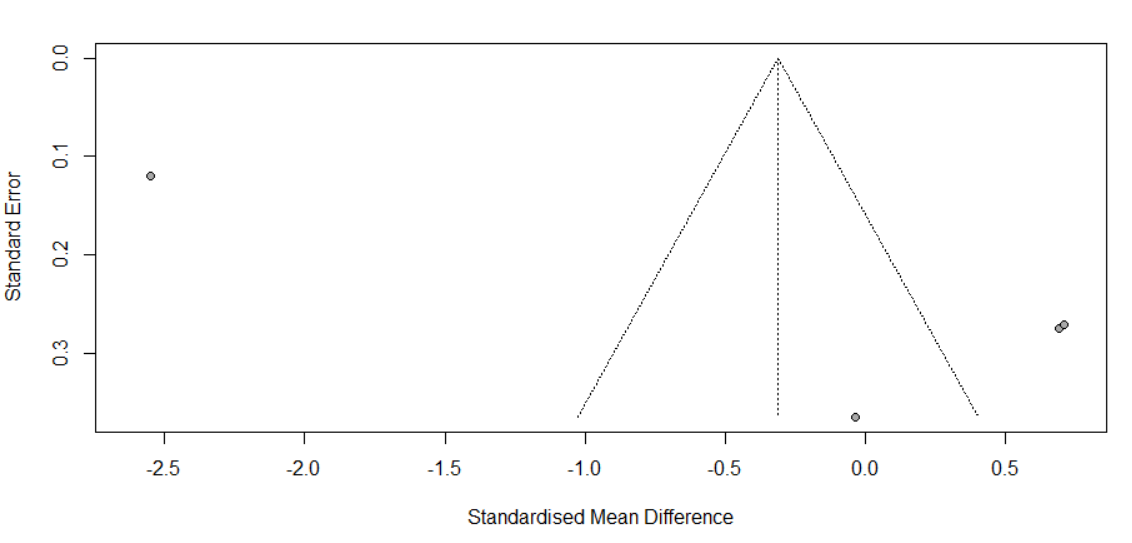
**

**TG >150 mg/dl**

**
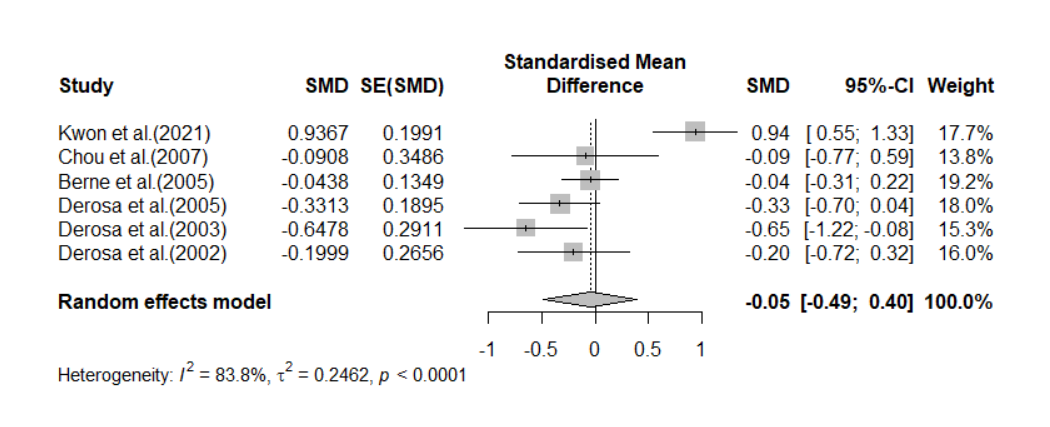
**

**
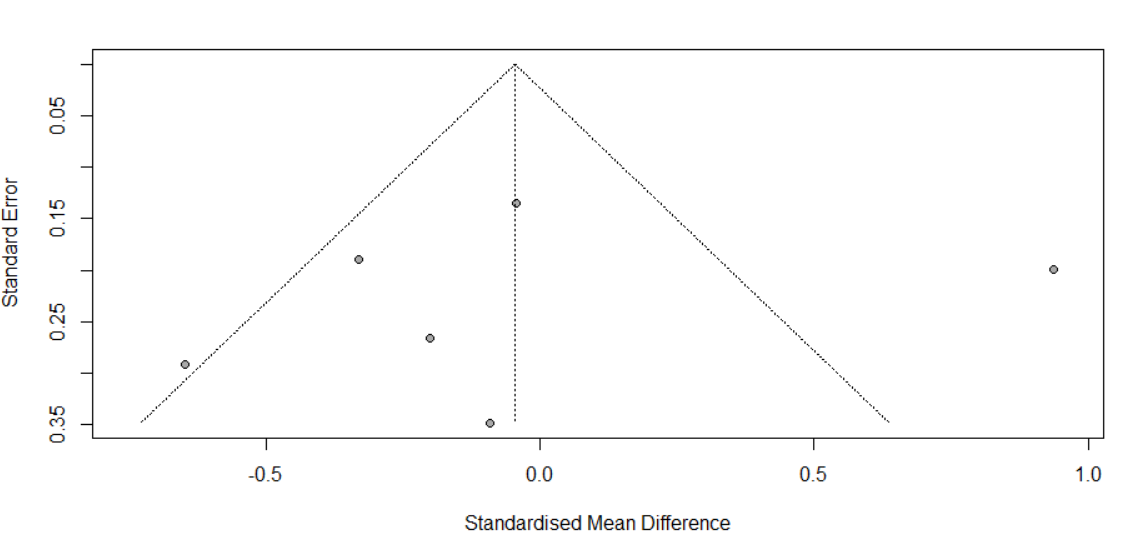
**

**TG <150 mg/dl**
