# Supplementary material for: Long‐Term Effects of Orlistat on Lipid Metabolism and Anthropometric Indices: A Meta‐Analysis of Clinical Trials
Source: J Obes. 2026 Feb 23;2026:9068305. doi: 10.1155/jobe/9068305 (PMC12927897; doi:10.1155/jobe/9068305)
Supplement: Supplementary file 3 — Supporting Information 3 Supporting Information 3: Intraclass correlation coefficient (ICC). [file JOBE-2026-9068305-s003.docx]

| Outcome | Consensus cells (N) | Paired cells for ICC (N) | Both-found rate (%) | R1 missing vs consensus (N) | R2 missing vs consensus (N) | R1 mismatches >0.01 (N) | R2 mismatches >0.01 (N) | ICC(2,1) abs agreement | ICC 95% CI |
| --- | --- | --- | --- | --- | --- | --- | --- | --- | --- |
| Apolipoprotein | 32 | 28 | 87.5 | 4 | 0 | 1 | 0 | 0.99997 | 0.9999 to 1.0000 |
| BMI | 64 | 64 | 100 | 0 | 0 | 14 | 0 | 0.999733 | 0.9993 to 1.0000 |
| Fasting Blood Glucose | 96 | 92 | 95.8 | 4 | 0 | 24 | 24 | 0.999556 | 0.9993 to 0.9998 |
| Waist Circumference | 104 | 86 | 82.7 | 0 | 18 | 20 | 20 | 0.999894 | 0.9998 to 0.9999 |
| Waist-to-Hip Ratio | 36 | 36 | 100 | 0 | 0 | 0 | 0 | 1 | 1.0000 to 1.0000 |
| Triglyceride | 60 | 54 | 90 | 0 | 6 | 0 | 0 | 1 | 1.0000 to 1.0000 |
| HDL-C | 64 | 64 | 100 | 0 | 0 | 0 | 0 | 1 | 1.0000 to 1.0000 |
| LDL-C | 64 | 57 | 89.1 | 7 | 0 | 9 | 0 | 0.999141 | 0.9983 to 0.9997 |
| Total Cholesterol | 70 | 63 | 90 | 0 | 7 | 0 | 3 | 0.999942 | 0.9998 to 1.0000 |
| Overall (all continuous outcomes combined) | 590 | 544 | 92.2 | 15 | 31 | 68 | 47 | 0.999857 | 0.9998 to 0.9999 |

Supplementary materials 3. Table: Inter-reviewer reliability of continuous data extraction based on the intraclass correlation coefficient (ICC).

R1 = Reviewer 1; R2 = Reviewer 2
