# Supplementary material for: Long‐Term Effects of Orlistat on Lipid Metabolism and Anthropometric Indices: A Meta‐Analysis of Clinical Trials
Source: J Obes. 2026 Feb 23;2026:9068305. doi: 10.1155/jobe/9068305 (PMC12927897; doi:10.1155/jobe/9068305)
Supplement: Supplementary file 4 — Supporting Information 4 Supporting Information 4: Orlistat combination therapy analysis. [file JOBE-2026-9068305-s004.docx]

**Orlistat Combination Therapy Subgroup Analysis (Orlistat and lipid-lowering drugs vs Orlistat alone)**


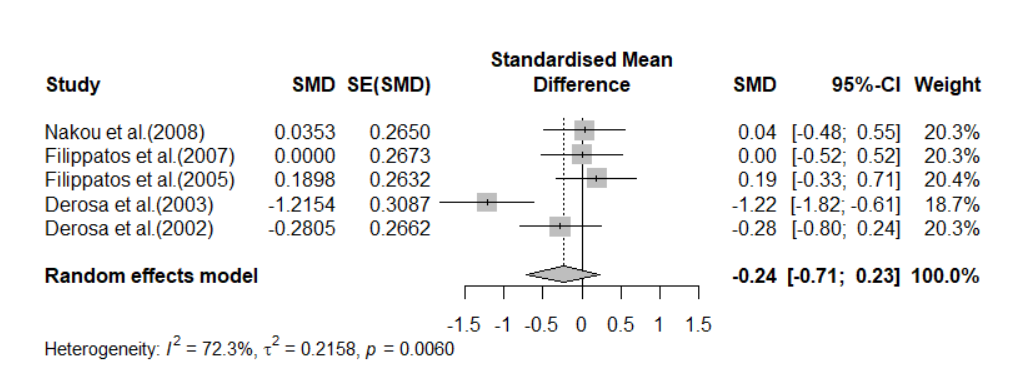


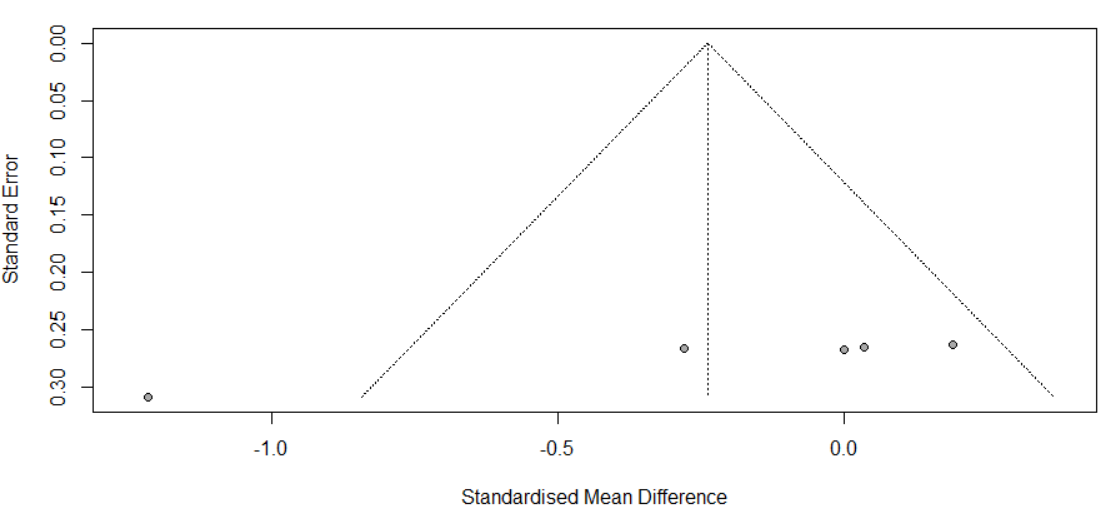


**BMI**

**
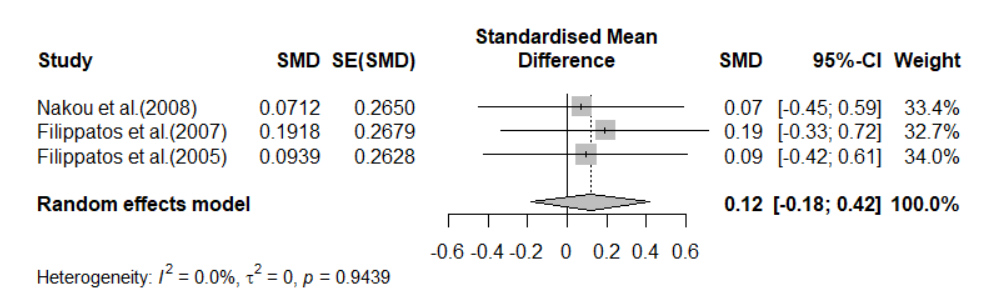
**

**
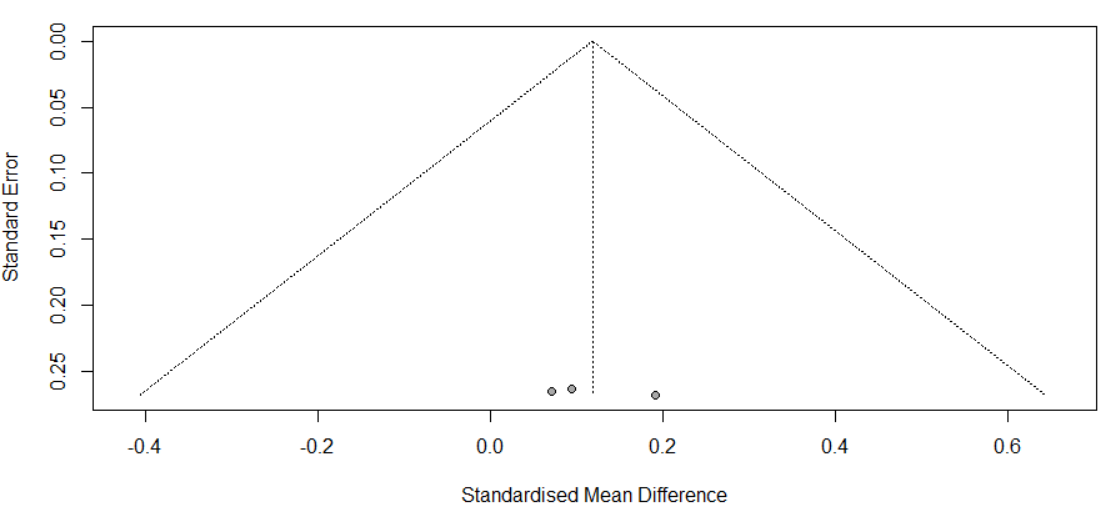
**

**Waist Circumference (WC)**

**
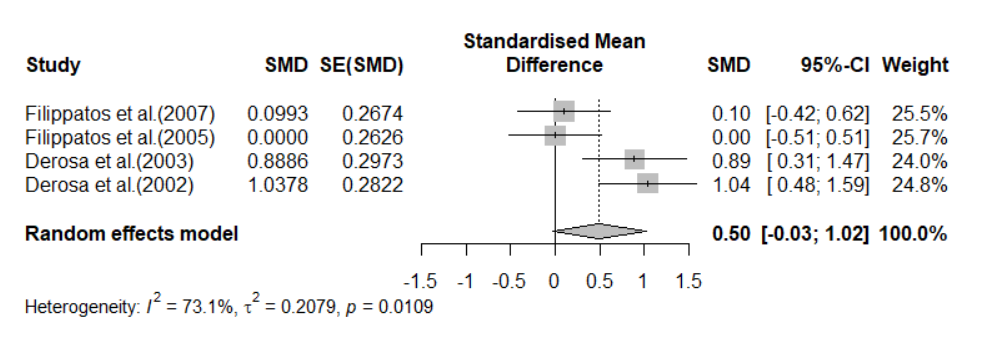
**

**
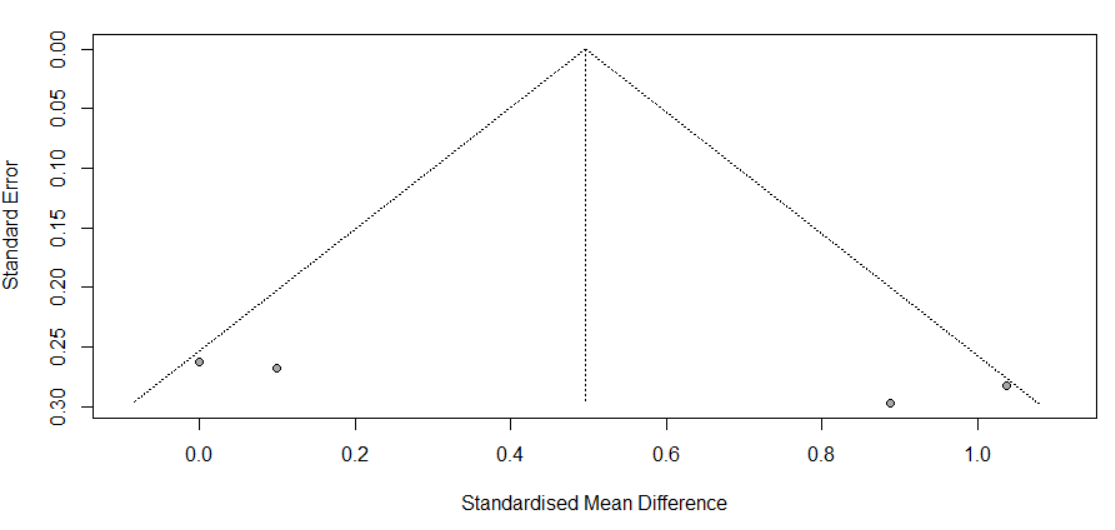
**

**HDL-C**

**
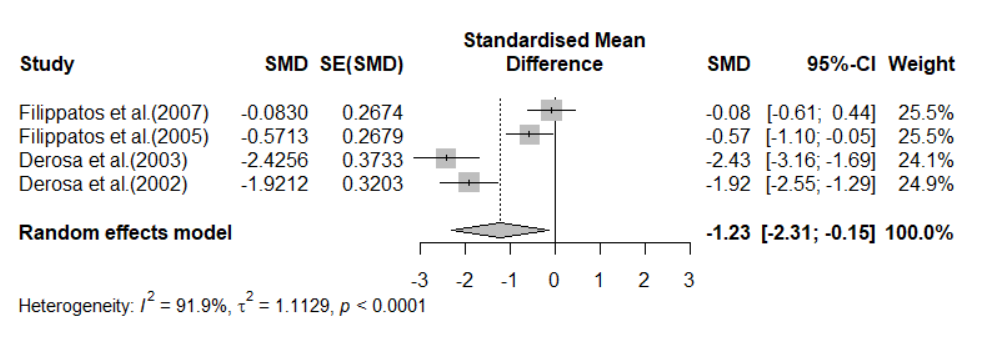
**

**
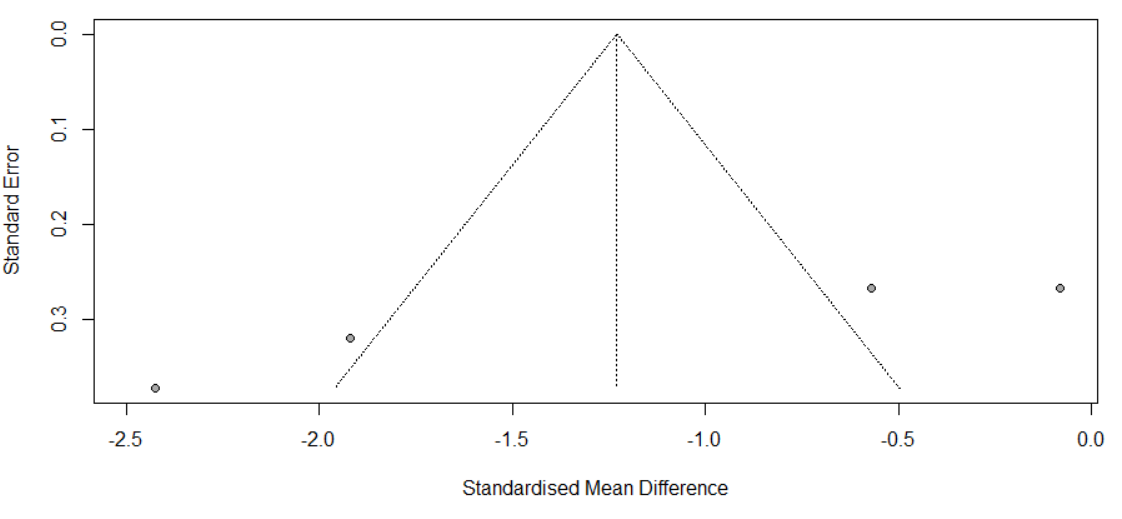
**

**LDL-C**

**
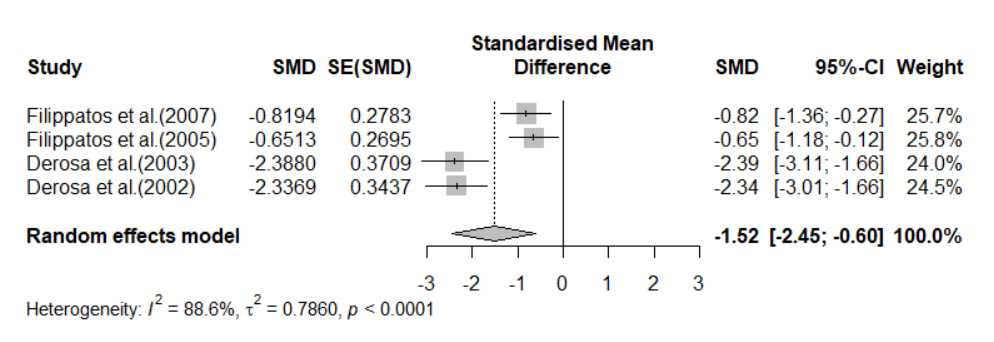
**

**
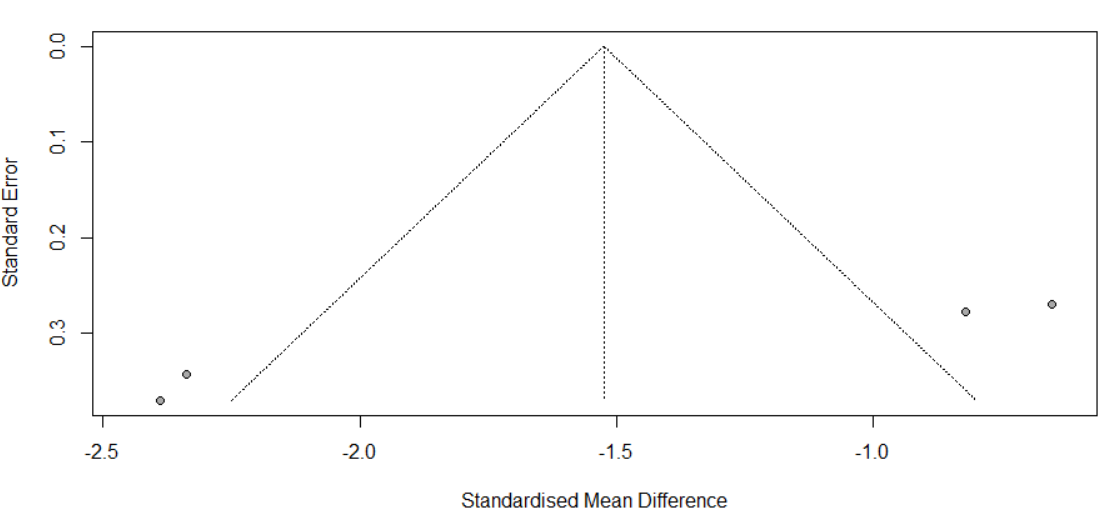
**

**Total Cholesterol (TC)**

**
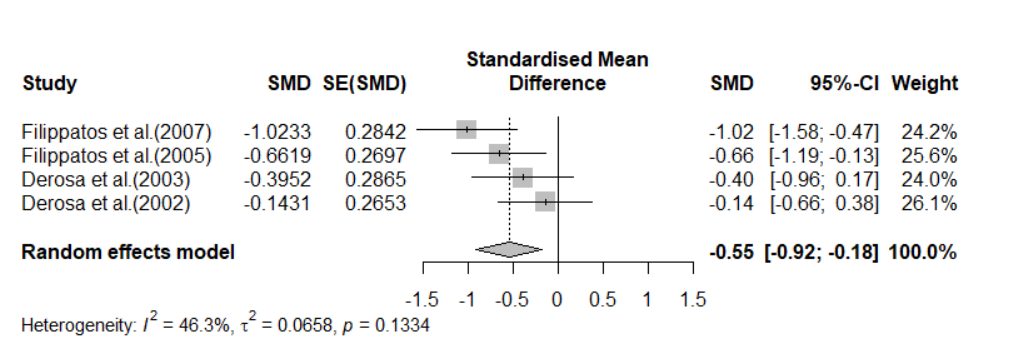
**

**
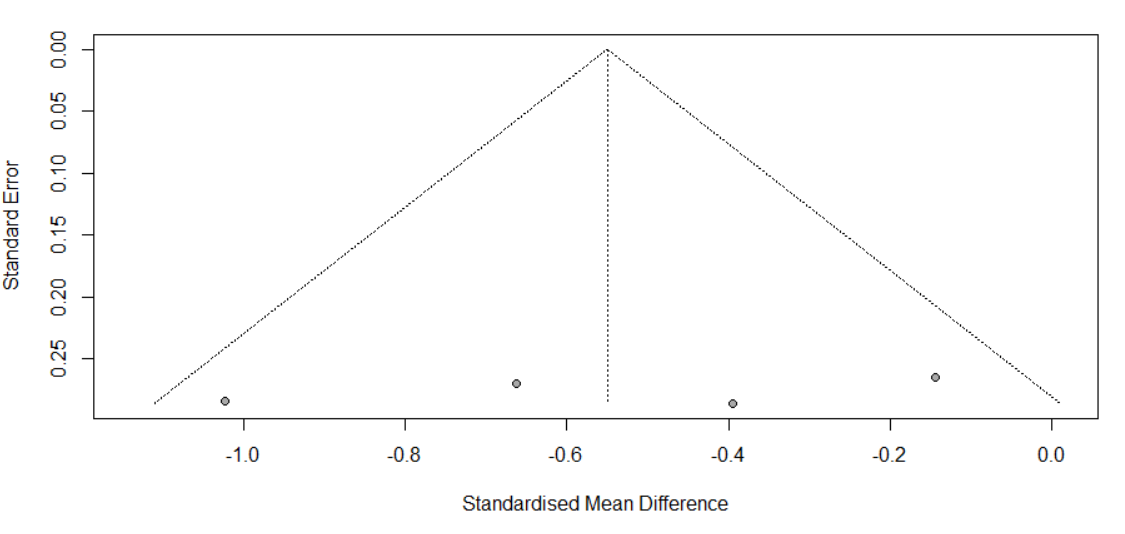
**

**Triglyceride (TG)**
